# Altered expression of vesicular trafficking machinery in prostate cancer affects lysosomal dynamics and provides insight into the underlying biology and disease progression

**DOI:** 10.1038/s41416-024-02829-x

**Published:** 2024-08-31

**Authors:** Bukuru D. Nturubika, Carlos M. Guardia, David C. Gershlick, Jessica M. Logan, Carmela Martini, Jessica K. Heatlie, Joanna Lazniewska, Courtney Moore, Giang T. Lam, Ka L. Li, Ben S-Y Ung, Robert D. Brooks, Shane M. Hickey, Andrew G. Bert, Philip A. Gregory, Lisa M. Butler, John J. O’Leary, Douglas A. Brooks, Ian R. D. Johnson

**Affiliations:** 1 Mechanisms in Cell Biology and Diseases Research Group, Clinical and Health Sciences, University of South Australia, Adelaide, SA 5000 Australia; 2 Placental Cell Biology Group, National Institute of Environmental Health and Science, National Institutes of Health, Research Triangle Park, NC 27709 USA; 3 Cambridge Institute for Medical Research, University of Cambridge, Cambridge, UK; 4 Quality Use of Medicines and Pharmacy Research Centre, University of South Australia City East Campus, Frome Rd, Adelaide, SA 5000 Australia; 5 Centre for Cancer Biology, University of South Australia, Adelaide, SA 5000 Australia; 6 South Australian ImmunoGENomics Cancer Institute and Freemasons Centre for Male Health and Wellbeing, University of Adelaide, Adelaide, SA 5000 Australia; 7 Solid Tumour Program, Precision Cancer Medicine theme, South Australian Health and Medical Research Institute, Adelaide, SA 5000 Australia; 8 Department of Histopathology, Trinity College Dublin, Dublin, Dublin 8 Ireland

**Keywords:** Lysosomes, Prostate cancer, Prostate cancer

## Abstract

**Background:**

This study focuses on the role of lysosomal trafficking in prostate cancer, given the essential role of lysosomes in cellular homoeostasis.

**Methods:**

Lysosomal motility was evaluated using confocal laser scanning microscopy of LAMP-1-transfected prostate cells and spot-tracking analysis. Expression of lysosomal trafficking machinery was evaluated in patient cohort databases and through immunohistochemistry on tumour samples. The roles of vesicular trafficking machinery were evaluated through over-expression and siRNA. The effects of R1881 treatment on lysosome vesicular trafficking was evaluated by RNA sequencing, protein quantification and fixed- and live-cell microscopy.

**Results:**

Altered regulation of lysosomal trafficking genes/proteins was observed in prostate cancer tissue, with significant correlations for co-expression of vesicular trafficking machinery in Gleason patterns. The expression of trafficking machinery was associated with poorer patient outcomes. R1881 treatment induced changes in lysosomal distribution, number, and expression of lysosomal vesicular trafficking machinery in hormone-sensitive prostate cancer cells. Manipulation of genes involved in lysosomal trafficking events induced changes in lysosome positioning and cell phenotype, as well as differential effects on cell migration, in non-malignant and prostate cancer cells.

**Conclusions:**

These findings provide novel insights into the altered regulation and functional impact of lysosomal vesicular trafficking in prostate cancer pathogenesis.

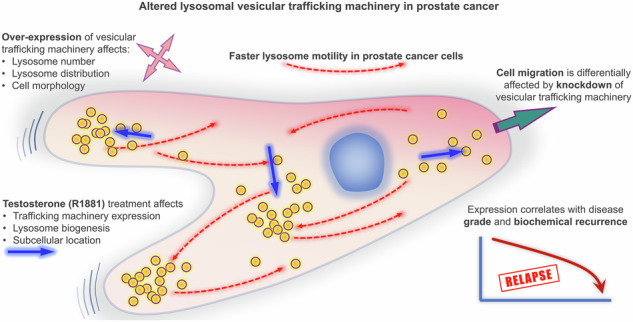

## Background

Prostate cancer is the second most diagnosed cancer in men worldwide, with more than 1.4 million new cases each year and over 375,000 patient deaths [1, 2]. Early detection and treatment can improve survival, but metastatic disease remains a significant challenge, with a five-year survival rate of only 31% [1]. To address this critical issue, there is an urgent need to understand the cellular biology of prostate cancer and the mechanisms of pathogenesis to facilitate the discovery of biomarkers for early detection and targeted therapeutic intervention.

Lysosomes are essential organelles for maintaining cellular homeostasis, immune function/inflammation, intracellular sensing and signalling, macromolecular degradation, and cellular recycling, and have a critical role in cancer biology. We have previously demonstrated that endosomal-lysosomal biogenesis is altered in prostate cancer [3–5], which led to the discovery of new biomarkers for disease detection in patient tissue [6–8]. However, the cellular mechanisms which underpin these alterations, and their functional impact, remain poorly understood.

Lysosome function is dependent upon the cellular distribution of the organelles [9]. For example, plasma membrane fusion, and exocytosis of hydrolases into the extracellular milieu requires anterograde lysosomal movement to the cell periphery [10]. Motor proteins such as kinesin-1 are critical to these processes; for example, knockdown of kinesin family member 5B (KIF5B) reduces lysosome-like mast cell granule exocytosis, which instead accumulate in perinuclear regions, indicative of disrupted anterograde trafficking [11]. The overexpression of KIF5B alongside co-factors kinesin light chain (KLC) and pleckstrin homology domain-containing family M member 2 (PLEKHM2), also known as SKIP, redistributes lysosomes to the cell periphery [12]. The movement and functional activity of lysosomes, as well as their distribution to the cell periphery, may be influenced by the disruption of Rab GTPases and the recruitment of vesicular trafficking machinery and transport of cargo. For example, the small GTPase ADP-ribosylation factor 6 (ARF6) has been implicated in prostate cancer invasion and metastasis by promoting matrix metalloproteinase (MMP) secretion and invadopodia formation [13]. Similarly, ADP ribosylation factor like GTPase 8B (ARL8B) is thought to play a crucial role in cancer cell invasion [14], with high levels of expression associated with poor prognosis in patients with breast cancer [15]. Moreover, invasion of prostate cells, mediated by cathepsin B secretion, has been shown to require anterograde trafficking [16].

Despite this circumstantial evidence, little is known about how the lysosomal trafficking machinery is regulated in prostate cancer cells, particularly during hormonal signalling by androgen, which is a critical factor for male development and prostate cancer pathogenesis [17]. A recent study identified the master regulator of lysosomal biogenesis, the lysosome associated transcription factor EB (TFEB), which is a transcriptional target of the androgen receptor (AR) in prostate cancer [18]. We hypothesised that androgen may regulate the expression of critical lysosomal vesicular trafficking machinery, enabling the coordination of lysosome biogenesis and organelle movement in response to androgen stimulation.

Herein, we show altered lysosome motility in prostate cancer cells, significantly altered expression of vesicular trafficking machinery in prostate cancer tissue that stratifies patients at risk of disease recurrence, significant effects of androgen on the expression of key lysosomal vesicular trafficking machinery and lysosomal biogenesis, and the ability to alter cell phenotypes through manipulation of vesicular trafficking gene or protein expression.

## Results

### Altered lysosomal organelle dynamics in prostate cancer cells

**Fig. 1:**
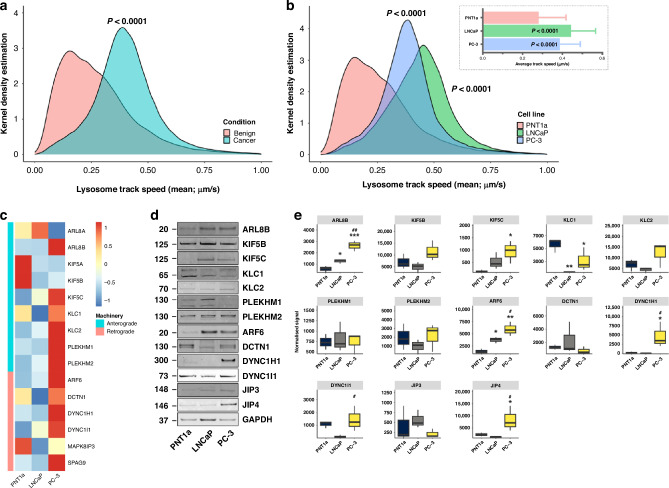
Prostate cancer cells display increased lysosome motility and significantly altered expression of lysosome-related trafficking machinery. **a** Kernel density estimations (KDE) from generalised linear mixed model (GLMM) of lysosome mean-track-speed (µm/s) pooled from non-malignant (pink; PNT1a, PNT2, PWR1E) and prostate cancer cell lines (turquoise; 22Rv1 LNCaP, DU-145, PC-3) transfected with LAMP1 revealed a significant increase in speed of lysosome movement in cancer cells (*P* < 0.0001). KDE is a nonparametric method to estimate the probability density function of a random variable based on kernels as weights. GLMM is an extension of the generalised linear model that allows for both fixed and random effects. **b** GLMM analysis of lysosome mean-track-speed (µm/s) revealed a significant increase in the speed of lysosome movement in LNCaP (blue; *P* < 0.0001) and PC-3 (green; *P* < 0.0001) cells compared to non-malignant PNT1a cells (pink); data was acquired from the lysosome track mean-speeds from *n* = 3 experiments, with 6–8 cells analysed per cell line per replicate, and the KDE plotted. **B inset**, the average mean-track-speed of lysosomes in LNCaP was significantly increased (*P* < 0.0001) and PC-3 (*P* < 0.0001) cells versus PNT1a (bars show standard deviation of the mean). **c** Heatmap representing the fold change of mRNA expression in LNCaP and PC-3 versus PNT1a cells, with grouping by anterograde (turquoise) or retrograde (pink) machinery function. **d**, **e** Western blot, and quantification of signal intensities relative to total protein load in PNT1a, LNCaP and PC-3 cells. Western blot images have been cropped from full-length membranes labelled by each antibody. Significance for LNCaP and PC-3 versus PNT1a: *P ≤ 0.05; **P ≤ 0.01; PC-3 versus LNCaP: #*P* ≤ 0.05; ##*P* ≤ 0.01.

The velocity of lysosome movement and end-stage localisation may play a crucial role in cancer pathogenesis, as these organelles facilitate inflammation, antigen presentation, signalling, plasma membrane repair, recycling during cell migration, exocytosis, and the uptake/degradation of cargo [19]. Here, we have investigated the spatiotemporal dynamics of lysosomes, using live-cell imaging of lysosomal associated membrane protein 1 (LAMP1)-GFP-transfected non-malignant prostate epithelial cell lines (PNT1a, PNT2 and PWR1E) and prostate cancer cell lines (22Rv1, LNCaP, DU145, and PC-3; Fig. 1a and Supplementary Videos 1). Prostate cancer cells exhibit significantly increased lysosome motility (*P* ≤ 0.0001) compared to non-malignant cells. The median lysosome track speed across all prostate cancer cells was significantly greater than the combined non-malignant cells. LNCaP and PC-3 cells, an androgen sensitive and castrate resistant prostate cell model, respectively, represent distinct subtypes of prostate cancer that enables exploration of the diversity of vesicular trafficking in prostate cancer. In comparison to PNT1a non-malignant cells, the mean-lysosome-track speed in both LNCaP and PC-3 cancer cells was significantly increased (*P* ≤ 0.0001; Fig. 1b and **inset**).

### Differential expression of anterograde and retrograde trafficking machinery may contribute to altered lysosomal trafficking

To quantify the changes in expression of the vesicular trafficking machinery in prostate cancer, we performed qPCR analysis on genes involved in anterograde and retrograde traffic in the prostate cancer cell lines LNCaP and PC-3, relative to the non-malignant PNT1a cell line (Fig. 1c and Supplementary Table 1). Significant alterations were observed in the expression of the kinesin-1 family members *KIF5A*, *KIF5B*, and *KIF5C*, which are involved in anterograde vesicular traffic. *KIF5A* and *KIF5B* were substantially downregulated in both cancer cell lines, while *KIF5C* had increased expression (See Supplementary Table 1 for fold change and *P*-values). *KLC1* and *KLC2*, kinesin light chain proteins, exhibited divergent expression. We also investigated genes critical for retrograde trafficking, such as dynactin subunit 1 (*DCTN1*) and dynein cytoplasmic 1 heavy chain 1 (*DYNC1H1*), which had reduced expression in LNCaP cells, but in contrast, increased expression of *DYNC1H1* in PC-3 cells. Furthermore, dynein cytoplasmic intermediate light chain 1 (*DYNC1I1*) had increased expression in both LNCaP and PC-3 prostate cancer cell lines (See Supplementary Table 1 for fold change and *P*-values).

To determine the relation of mRNA to protein expression, we conducted semi-quantitative analysis using Western blotting (Fig. 1d, e). There were significantly elevated amounts of ARL8B and ARF6 protein in both LNCaP and PC-3 cells relative to non-malignant controls. There was significantly increased KIF5C, DYNC1H1, and JNK-interacting protein 4 (JIP4) protein in PC-3 cells alone. KLC1 was significantly reduced in both cancer cell lines, while DYNC1I1 had notably higher protein amounts in PC-3 compared to LNCaP cells. ARL8A and KIF5A were not detected, presumably due to concentrations being below the detection threshold.

Our data revealed a complex and divergent landscape of alterations to anterograde and retrograde lysosomal vesicular trafficking machinery in prostate cancer cells. These findings suggest potential roles in the molecular pathogenesis of the disease and the changes in gene/protein expression may contribute to driving specific lysosomal trafficking to achieve functional outcomes in prostate cancer cells.

### Altered expression of genes involved in lysosomal trafficking was also observed in prostate cancer patient cohorts

**Fig. 2:**
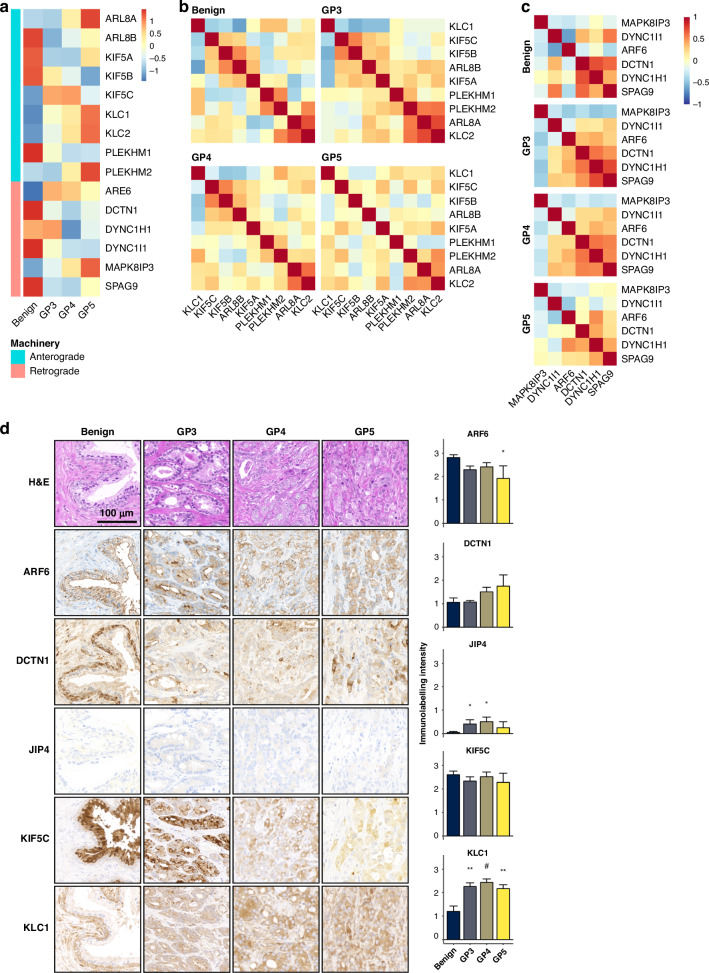
Altered expression of lysosome trafficking machinery in prostate cancer tissue. **a** mRNA expression heatmap comparing benign and prostate cancer tissue of GP3, GP4, and GP5, with genes grouped by anterograde (turquoise) or retrograde (pink) function. mRNA co-expression clustering of anterograde **b** and retrograde **c** vesicular trafficking machinery reveals distinct correlations in GP3 tissue compared to benign tissue. mRNA order is maintained across benign, GP3, GP4, and GP5 heatmaps. Scale reflects Pearson’s coefficient of correlation ( − 1 to 1). **d** Representative micrographs of immunohistochemical analysis and quantification and statistical analysis (GLMM) of signal (H-score) in benign and Gleason pattern 3, 4, and 5 (GP3, GP4, and GP5) prostate tissue (*n* = 9). Scale bar, 25 µm. *P ≤ 0.05; **P ≤ 0.01; #*P* ≤ 0.001; ##*P* ≤ 0.0001.

To substantiate the alterations in lysosomal trafficking machinery observed in vitro, we examined mRNA expression in publicly available datasets of patients with prostate cancer (Supplementary Tables 2–5). Using the Cancer Genome Atlas Prostate Adenocarcinoma (TCGA-PRAD [20]) dataset, we found significant alterations in lysosomal trafficking genes in prostate cancer patients that were consistent across Gleason patterns 3, 4, and 5 (GP3, GP4, and GP5), indicating a sustained disruption in lysosomal trafficking machinery during disease progression (Fig. 2a and Supplementary Tables 2, 5). Co-expression analysis of retrograde and anterograde vesicular trafficking genes revealed distinct expression patterns across different Gleason patterns (Figs. 2b, c; Supplementary Dataset 1). There is a potential shift in the regulatory dynamics of these genes during disease progression. Immunohistochemical analyses on representative benign and prostate cancer tissues (Fig. 2d and Supplementary Fig. 1) corroborated these transcriptional alterations at the protein level. Altered membrane localisation and labelling patterns of several genes were observed, suggesting a potential contribution of altered vesicular trafficking machinery to changes in lysosome localisation and function.

### Lysosomal trafficking genes as predictors of prostate cancer recurrence

**Fig. 3:**
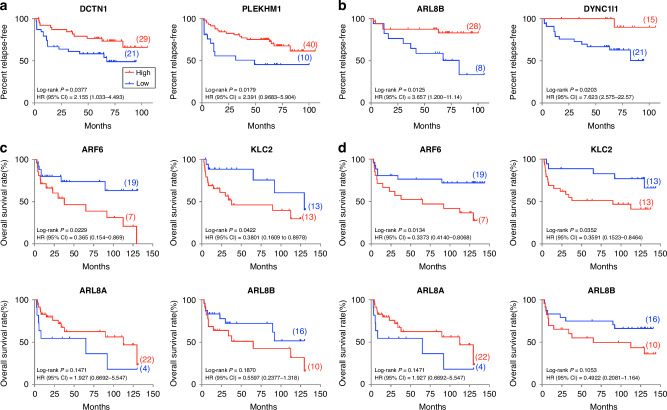
Kaplan-Meier analysis based on K-means clustering of mRNA expression demonstrates the stratification of prostate cancer patients at risk of BCR or CR by lysosome vesicular trafficking machinery expression. **a** Analysis of the Glinsky Cohort (*n* = 79) by K-means clustering. **b** Stratification of patients from Glinsky cohort with PSA < 10 ng/mL (*n* = 50). Analysis of BCR **c** and CR **d** endpoints in the APCB cohort (*n* = 47). Patients with lower expression are represented by blue line, higher expression by red line.

Differential expression of endosome and lysosome-related mRNA has been identified as a potential predictor of biochemical recurrence (BCR [4, 6, 7]). Building on our earlier findings, which highlighted the downregulation of *ARL8B* and *DYNC1I1* in patient tissues but not in an in vitro setting, we further assessed the potential of lysosomal trafficking genes as predictors of BCR and clinical recurrence (CR). Using the Glinsky cohort, patients were classified based on mRNA expression levels (Fig. 3). Lower expression of *DCTN1* and *PLEKHM1* were linked to BCR, indicating potential clinical recurrence and worse prognosis (Fig. 3a). Lower expression of *ARL8B* and *DYNC1I1* stratified patients at risk of BCR when initial pre-prostatectomy PSA was less than 10 ng/mL (Fig. 3b). An additional cohort from the Australian Prostate Cancer BioResource (APCB) was analysed (Fig. 3c, d). Higher expression of *ARF6* and *KLC2* were identified as predictors of BCR and CR. Low expression of *ARL8A* indicated a shorter time to both BCR and CR. While reduced *ARL8B* expression was associated with shorter time to CR in the Glinsky cohort and in patients with PSA < 10 ng/mL, the APCB cohort revealed only a trend for higher expression leading to BCR or CR. These findings suggest that lysosomal trafficking genes, including *DCTN1*, *PLEKHM1*, *ARL8B*, and *DYNC1I1*, could serve as potential predictors of patient outcomes, highlighting the nuanced but potentially decisive role of trafficking machinery in prostate cancer progression and recurrence.

### Altered regulation of vesicular trafficking machinery changes lysosome positioning and dynamics in prostate cancer cells

**Fig. 4:**
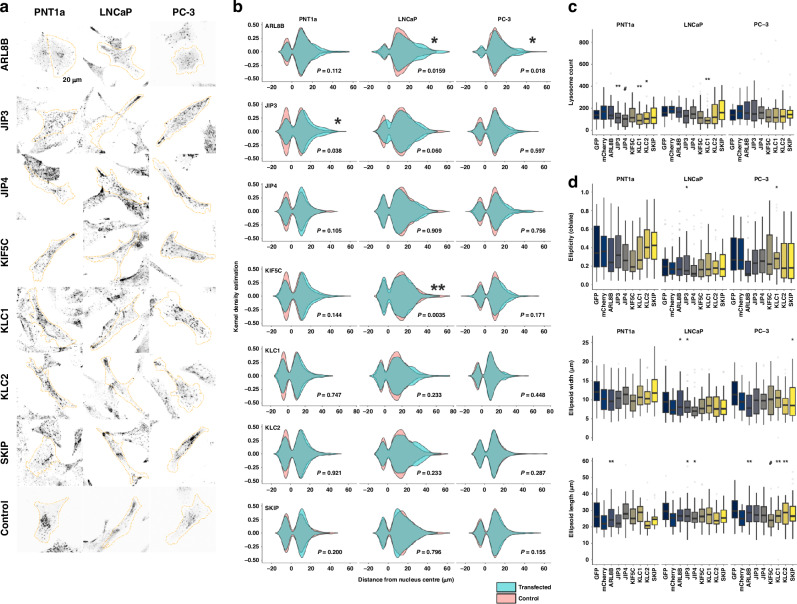
Altered lysosome location and cell morphology induced by over-expression of lysosomal vesicular trafficking machinery in prostate cells. **a** Representative micrographs showing PNT1a, LNCaP, and PC-3 cells over-expressing lysosomal vesicular trafficking machinery. Scale bar, 20 µm. **b** Quantification of the shortest distance between lysosome centre to nucleus surface by KDE and GLMM, revealing altered lysosome distribution in cells over-expressing specific vesicular trafficking machinery (turquoise) compared to controls (pink). **c, d** Boxplots depicting quantification of lysosome number, ellipticity (oblate), cell width and length in cells over-expressing trafficking machinery. Data were collected from approximately 30 cells across three biological replicates. *P ≤ 0.05; **P ≤ 0.01; **#***P* ≤ 0.01; **##***P* ≤ 0.001.

Building upon our findings of altered expression for genes/proteins involved in lysosomal trafficking in prostate cancer patient cohorts, we explored the functional implications of these molecules in cells. Employing transient transfection approaches in prostate cell lines we revealed an impact on lysosome positioning and number of organelles (Fig. 4, Supplementary Table 6). JIP3 overexpression in PNT1a cells resulted in a significant redistribution of lysosomes (*P* = 0.038; Fig. 4a, b) and a marked decrease in the number of lysosomes (*P* ≤ 0.01; Fig. 4c; Supplementary Table 6b). In LNCaP cells, we observed that overexpression of JIP3 and JIP4 was concomitant with marked alterations in cellular morphology, notably an increase in ellipticity via a reduction in cell length (*P* ≤ 0.05; Fig. 4d; Supplementary Tables 6c, 6e). Lesser but similar effects on the number of lysosomes were observed for increased expression of KLC1 and KLC2 (Fig. 4; Supplementary Table 6b). In both LNCaP and PC-3 cancer cells, increased ARL8B expression induced a notable perinuclear-to-peripheral distribution shift in lysosomal positioning (Fig. 4a, b; *P* = 0.0159, *P* = 0.018 respectively), a phenomenon not observed in non-malignant PNT1a cells (*P* = 0.112). The morphological changes in response to increased ARL8B expression included an increase in cell width in LNCaP cells and cellular elongation in PNT1a and PC-3 cells (Fig. 4d, Supplementary Table 6d, 6e).

There was concordance between mRNA expression datasets (Fig. 1c, Supplementary Table 1), and upregulated genes/proteins in specific cell lines (e.g., for ARL8B expression in LNCaP and PC-3 cells, and KIF5C expression in LNCaP cells) and the effects on lysosomal dynamics (Supplementary Table 6). This suggested that the intrinsic molecular architecture of each cell line may precondition its sensitivity to manipulations in lysosomal vesicular trafficking machinery. The dynamic balance between anterograde and retrograde vesicular trafficking mechanisms may be integrally involved in the pathogenesis of prostate cancer, raising the question of how this might be influenced by hormone changes, which are known to be involved in prostate cancer progression.

### Altered lysosomal biogenesis and trafficking elicited by synthetic androgen R1881 in hormone-sensitive prostate cells

**Fig. 5:**
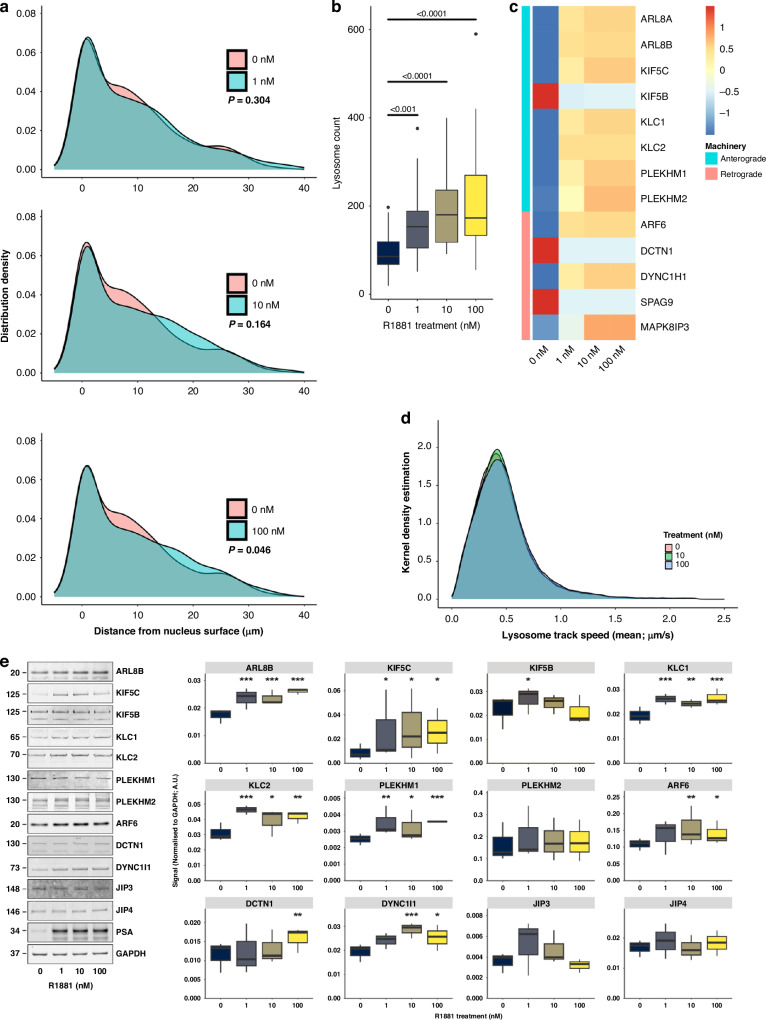
Hormone-sensitive cells exhibit a spatiotemporal response of lysosomes to 48 h R1881 treatment. **a** KDE and statistical analysis (GLMM) reveal a significant change (*P* = 0.046) in lysosome distribution in R1881-treated LNCaP cells (100 nM; turquoise) versus untreated cells (0 nM; pink). **b** Lysosome biogenesis increases significantly in response to R1881 treatment. Data were obtained from three biological replicates where 10 cells per treatment have been imaged, per replicate and statistical analysis performed using GLMM. **c** Heatmap of the relative mRNA fold change from RNASeq analysis of LNCaP cells treated with R1881. **d** Kernel density estimation of lysosome mean-track-speed (µm/s) in LNCaP cells treated with 0 nM (pink), 10 nM (green) or 100 nM (blue) R1881, from eight biological replicates with 6–10 cells captured per treatment, per replicate. **e** Representative Western blots, and quantification from three biological replicates of signal intensities relative to GAPDH in LNCaP cells treated 1, 10 and 100 nM R1881 compared to untreated cells. Western blot images have been cropped from full-length gels and membranes labelled by each antibody. Statistical analysis performed using GLMM: *P ≤ 0.05; **P ≤ 0.01; ***P ≤ 0.001.

In situ expression analyses in normal and cancer tissues demonstrated a statistically significant correlation between androgen receptor (AR) expression and genes involved in lysosomal vesicular trafficking (Fig. 2b, c). This association indicated a potential hormone regulated facet of lysosomal biology, which could be important for the understanding of castration-resistant prostate cancer (CRPC). The synthetic androgen R1881 was administered to LNCaP prostate cells, leading to a dose-dependent alteration in lysosomal distribution (Fig. 5A**;** Supplementary Fig. 2) and a significant increase in lysosomal biogenesis (Fig. 5d). Further investigation revealed differential expression of several genes/proteins involved in lysosomal vesicular trafficking upon R1881 treatment (Fig. 5c, e; Supplementary Tables 7–9). Notably, protein levels of ARF6, ARL8B, DCTN1, KIF5B, KIF5C, KLC1, KLC2, DYNC1I1, and PLEKHM1 were substantially elevated in LNCaP cells, despite reductions in mRNA expression for KIF5B and SPAG9. R1881 treatment did not consistently alter lysosomal motility (Fig. 5d). The findings suggest that androgen may significantly influence lysosomal biogenesis and vesicular trafficking machinery in prostate cancer pathogenesis or its treatment by androgen deprivation therapy.

### Altered expression of lysosome trafficking machinery differentially affects the migration of prostate cells

**Fig. 6:**
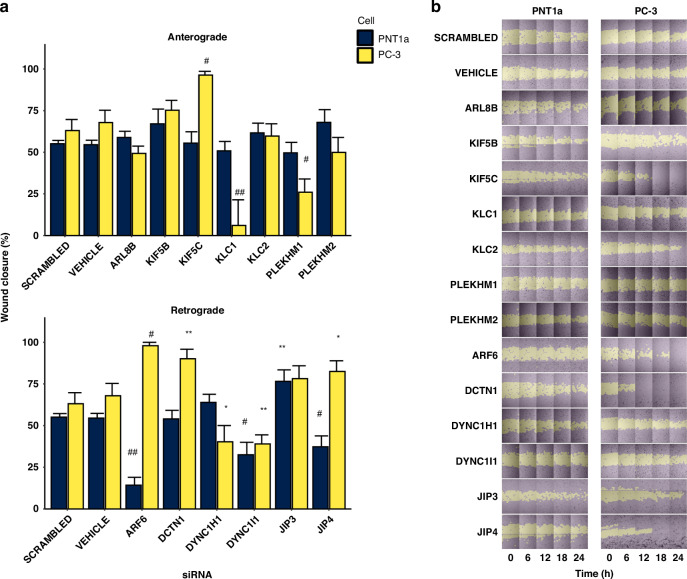
The migration potential of PNT1a non-malignant and PC-3 malignant prostate cancer cells is affected by siRNA knockdown of lysosomal vesicular trafficking machinery. **a** Quantification of wound closure after 24 h, subsequent to 72 h siRNA treatment. **b** Representative images of wound closure rate monitored for 24 h. Data were presented as the mean percentage of wound closure at 24 h from at least three wound areas per well, and two wells for each independent biological replicate (*n* = 3). Error bars indicate the standard error of the mean (SEM). Statistical significance of migration differences between siRNA treated cells relative to scrambled controls was determined by LME. *P ≤ 0.05; **P ≤ 0.01; #*P* ≤ 0.001; ##*P* ≤ 0.0001.

To further investigate the mechanistic implications of altered lysosomal vesicular trafficking, we employed siRNA-mediated gene silencing targeting a range of trafficking proteins (Fig. 6; Supplementary Table 10 and Supplementary Fig. 2) and assessed the impact on cell migration. The depletion of *ARF6* and *JIP4* significantly augmented the migratory capabilities of malignant PC-3 cells (*P* ≤ 0.001 and *P* ≤ 0.05, respectively), while concomitantly inhibiting cell migration in non-malignant PNT1a cells (*P* ≤ 0.0001). These findings delineate a stark dichotomy in how ARF6 and JIP4 influence cell migration, which may be contingent upon the malignant status of the cells or regulatory components of the lysosomal vesicular machinery. Conversely, *JIP3* knockdown significantly increased the migratory capacity of non-malignant cells (*P* ≤ 0.01) though an increase observed in PC-3 cells was not statistically significant. *DCTN1* and *KIF5C* knockdown selectively enhanced the migration of PC-3 cells (*P* ≤ 0.05), whereas the depletion of *KLC1*, *PLEKHM1* and *DYNC1H1*, selectively inhibited migration in PC-3 cells (*P* ≤ 0.001, *P* ≤ 0.01 and *P* ≤ 0.05, respectively). Notably, the knockdown of *DYNC1I1* had a universal inhibitory effect on migration in both non-malignant PNT1a cells (*P* ≤ 0.01) and PC-3 (*P* ≤ 0.01) cancer cells. Technical limitations prevented us from assessing the function of this trafficking machinery in LNCaP cells.

### EMT protein expression is modulated by altered trafficking machinery

**Fig. 7:**
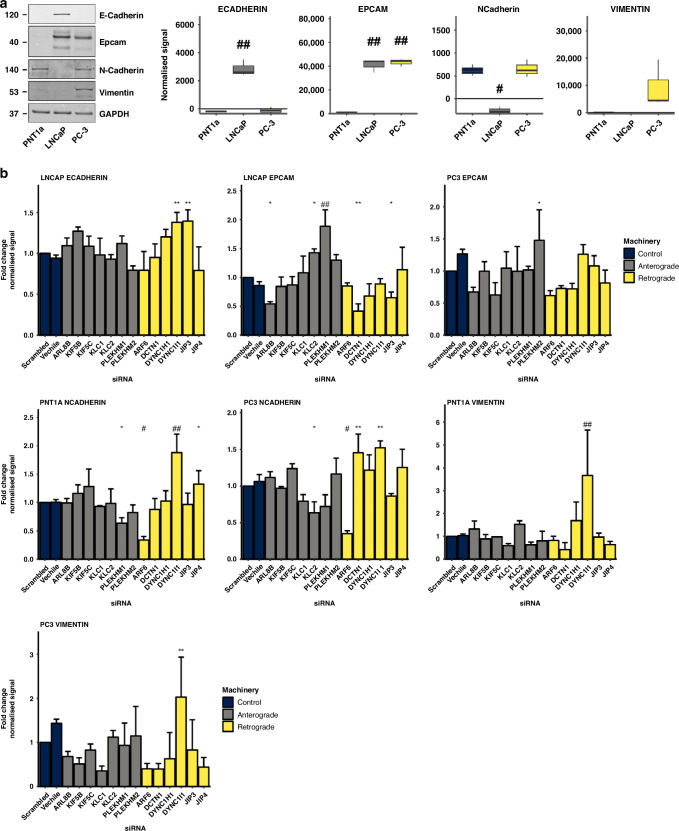
Expression of EMT proteins is modulated by lysosome trafficking machinery loss of function. **a** Representative Western blots, and quantification of EMT protein expression in PNT1a, LNCaP and PC-3 cells from three biological replicates. **b** Quantification of EMT protein expression in PNT1a, LNCaP and PC-3 cells from three biological replicates of lysosome trafficking machinery siRNA knockdown experiment. Data are presented as mean signal intensity and fold changes normalised to GAPDH and error bars indicate the standard error of the mean (SEM). Statistical significance of signal intensity differences between siRNA treated cells relative to scrambled controls was determined by LME. *P ≤ 0.05; **P ≤ 0.01; #*P* ≤ 0.001; ##*P* ≤ 0.0001.

The influence of trafficking machinery loss of function on the migration and gain of function on the morphology of prostate cancer cells, suggests an involvement of lysosome trafficking machinery in modulating processes that mediate the migration of prostate cancer cells, and specifically epithelial to mesenchymal transition (EMT). Using siRNA-mediated gene knock-down, we evaluated whether the loss of function of lysosome trafficking machinery affects epithelial to mesenchymal transition (EMT) protein expression (Fig. 7; Supplementary Fig. 3). We characterised the expression of the epithelial markers E-cadherin and EpCAM, as well as the mesenchymal markers N-cadherin and Vimentin in PNT1a, LNCaP and PC-3 cells. E-cadherin expression was only observed in LNCaP cells (Fig. 7A). EpCAM protein was significantly overexpressed in LNCaP and PC-3 relative to PNT1a cells (Fig. 7A). N-Cadherin was significantly decreased in LNCaP cells relative to PNT1a cells. PC-3 cells had significantly higher amounts of Vimentin relative to PNT1a cells. The EMT marker protein expression profile aligns with a different epithelial and mesenchymal phenotype in LNCaP and PC-3 cells. Remarkably, the knockdown of DYNC1I1 promoted significant upregulation of EMT proteins in all cell lines whilst the knockdown of other trafficking machinery was selective (Fig. 7B; Supplementary Fig. 4). The knockdown of JIP3 significantly increased the expression of E-cadherin in LNCaP cells. The expression of EpCAM was reduced upon ARL8B, DCTN1 and JIP3 knockdown, and increased upon KLC2 and PLEKHM1 knockdown in LNCaP cells. In PC-3 cells, a reduction of EPCAM was observed upon PLEKHM2 knockdown. Knockdown of ARF6 and PLEKHM1 reduced the expression of N-Cadherin in PNT1a and PC-3 cells.

## Discussion

Lysosomes are dynamic organelles that have a fundamental role in cell homeostasis [21], and their biogenesis is altered in prostate cancer cells [3, 4]. Lysosomes have functional roles in energy sensing/nutrient uptake, intracellular degradation, immune response, cellular signalling/migration [10], which requires precise control of their spatial and temporal dynamics, by vesicular trafficking machinery [22]. In our study, we discovered significant changes in the expression of retrograde and anterograde vesicular trafficking machinery, accompanied by alterations in lysosome motility in prostate cancer cells. Retrograde and anterograde lysosomal trafficking machinery work dynamically to control the directional flow of lysosomal traffic. Therefore, the observed alterations in lysosomal genes/protein expression could potentially impact on the balance between anterograde and retrograde transport, thereby affecting lysosomal trafficking and function. These alterations affected lysosomal number and positioning, which subsequently influenced cell morphology and cell migration. Notably, the expression of lysosomal vesicular machinery was found to be responsive to treatment with the androgen analogue R1881. This connection between androgen and altered lysosomal vesicular trafficking may hold importance in lethal castration-resistant prostate cancer (CRPC), potentially providing an alternative target for therapeutic intervention.

We found that patients with higher ARF6 expression correlated with increased potential for biochemical or clinical recurrence. The small GTPase ARF6 has been implicated in the regulation of lysosome exocytosis and cancer cell invasion [13, 23] and this has previously been reported in patient cohorts of prostate cancer [24]. ARF6 is a membrane associated protein, which can interact with kinesin-1 and dynactin/dynein via JIP3/JIP4, to promote exocytosis, which is critical for normal prostate gland function. Thus, the changes in JIP3 and JIP4 localisation we observed in prostate tumours may cause alterations to cell surface delivery and recycling pathways, which can in turn affect cargo delivery, such as the metalloproteinase MMP14 that are used to degrade extracellular matrix [25]. The upregulated expression of ARF6 may therefore result in increased exocytosis of membrane-type matrix metalloproteinases, consistent with promoting cancer cell invasion.

The migratory potential of prostate cells may be further influenced by direct alterations to the dynein motor proteins by modulation of dynein subunit expression, which affects retrograde trafficking. However, alterations to either the kinesin motor protein or kinesin adaptors, responsible for anterograde trafficking, was sufficient to significantly affect the migration of only PC-3 cancer cells, but not non-malignant cells. Conversely, JIP4 knockdown had differential effects on the migration rates of non-malignant (reducing) or cancer cells (increasing). JIP4 is required to recruit lysosomes for retrograde transport under the regulation of ARF6 [26]. In addition, JIP3 knockdown, which also acts downstream of ARF6, increased migration rates in non-malignant and cancer cells. PC-3 cells express significantly higher levels of JIP4 and ARF6, and significantly lower levels of JIP3 compared to non-malignant PNT1a cells. These altered levels of expression could influence how each cell type responds to gene knockdown. Beyond their role in lysosome vesicular trafficking, ARF6 and JIP4 also control the trafficking of recycling endosomes, during, for example, cell abscission in cytokinesis in conjunction with kinesin-1 and the dynactin complex [27]. We did not observe changes to prostate cell phenotype or migration from KIF5B knockdown, yet KIF5C silencing significantly increased migration of prostate cancer cells. This observation aligns with previous studies that have reported dysregulation of KIF5 expression in various types of cancer and its association with cancer progression [28, 29]. The complexity of vesicular trafficking is exemplified by the differential effects of KIF5B knockdown which enhances cell death in HeLa cells compared to MCF-7 cells, yet both cells exhibit similar levels of expression [30]. These findings underscore the intricate role of anterograde and retrograde machinery on cell migration, and the multifaceted effects of vesicular trafficking machinery that may extend beyond lysosome trafficking, on prostate cancer pathogenesis.

Lysosome distribution is significantly affected by the expression of ARL8B/ KIF5C anterograde and JIP3 retrograde molecular machinery. Interestingly, both PNT1a and LNCaP cells expressed comparatively lower levels of the adaptor protein ARL8B, yet PNT1a remained unaffected by ARL8B over-expression, which suggests alternate regulation of anterograde trafficking in the cancer cells, with distribution of lysosomes to the cell periphery. In addition, the over-expression of the motor protein KIF5C in LNCaP cells, which is androgen regulated, also resulted in lysosome distribution changes to the perinuclear region of the cancer cells. Increased expression of the adaptor protein JIP3 unexpectedly resulted in the peripheral distribution of lysosomes in PNT1a cells, which may be explained by disruption of dynein processivity as previously shown with the co-factor dynactin [12, 31]. JIP3 mutants that cannot bind dynein can still promote anterograde traffic to the cell periphery, but leucine zipper domain JIP3 mutants that block kinesin binding stop this anterograde traffic [32]. Moreover, JIP3 and its paralog JIP4 is also known to interact with kinesin motors to promote anterograde transport of organelles, suggesting that increased JIP3/JIP4 in the cancer cells may promote increased recruitment of the kinesin anterograde machinery rather than the dynein retrograde machinery [32]. In addition, increased JIP3 expression promoted a significant peripheral redistribution of lysosomes in non-malignant cells, but not in the cancer cells which already exhibit peripheral lysosome positioning [3] and high amounts of JIP4 expression that would also be aided by the increases in kinesin-1 (KIF5C, KLC2) and regulatory protein ARF6 expression. Notably, we have only analysed a comparatively small sample of trafficking related genes/proteins, and whilst the data present some evidence of a complex control system for dynamic trafficking in prostate cells, further analysis of other components and regulatory aspects of the lysosomal trafficking machinery are required to understand the trafficking process in non-malignant and prostate cancer cells.

We had observed significant positive and negative co-expression of anterograde as well as retrograde vesicular trafficking machinery; for example, with the transition from benign to GP3 tumour morphology. These changes suggest a potential role for alterations in expression and the pathogenesis of the disease, whist many non-significant correlations with GP5 tumours could indicate a breakdown in the typical regulatory relationships, further compounding a deregulated state in the vesicular trafficking machinery in high-grade disease. Notably, *MAPK8IP3* (JIP3) had a significant negative correlation with retrograde trafficking genes, whilst *SPAG9* (JIP4) had a significant positive correlation, in GP3 and GP4 tumours. Whilst JIP3 and JIP4 have similar roles in lysosomal trafficking, as shown in neurons [33], these differences in co-expression may suggest a preference to specific vesicular trafficking events; particularly considering recent evidence that the loss of JIP3 reduces endocytic uptake into neurons [34].

The dysregulation of ARL8B vesicular trafficking machinery has been implicated in therapeutic resistance and cancer pathogenesis [15], and is associated with an increased risk of disease recurrence in patients with prostate cancer. ARL8B is a small GTPase that plays a critical role in autophagy and the secretion of MMPs that can facilitate cancer progression [14, 35]. Changes to the expression of subunits of this machinery have previously been shown to affect lysosome positioning/location [35]; where the anterograde adaptor links lysosomes to kinesin and plays a significant role in retrograde lysosome movement [36]. Reductions in ARL8 expression may inhibit PLEKHM2 activation, preventing kinesin1 binding to lysosome cargo [37]. The regulation of these proteins is complex and involves multiple layers of control, including autoinhibition mechanisms and the significant changes we have observed in prostate cancer suggest that this may be directly involved in the pathogenic process.

Dysregulated expression of DCTN1 has been reported in several cancers [38, 39], and here we revealed altered DCTN1 expression in prostate cancer tumours, suggesting its potential involvement in the pathogenic process. DCTN1 can form fusions with anaplastic lymphoma kinase (ALK), a tyrosine kinase that can drive growth and progression of many solid tumours and requires lysosomal degradation to attenuate its signalling [40–42]. Interestingly, the over-expression of DCTN1 disrupts its association with dynein and causes displacement of lysosomes to the cell periphery [12, 31]. Furthermore, DCTN1 over-expression impairs lysosome maturation and fusion with late endosomes and multivesicular bodies, which in turn decreases lysosomal degradative enzyme activity [43]. Whilst ALK fusions are associated with poor prognosis, they have not been extensively studied in prostate cancer. Interestingly, KIF5B, KLC1 and PLEKHM2 that mediate anterograde lysosome trafficking have also been identified as ALK fusion partners [44]. ALK fusions with DCTN1 or other lysosome trafficking proteins may impair lysosomal traffic and maturation, promoting increased ALK signalling through perturbed lysosomal degradation, facilitating prostate cancer development.

The positive correlation between androgen receptor expression and most of the lysosomal vesicular trafficking genes in low grade tumours, but not in higher-grade disease, suggested involvement in early-stage pathogenesis, where androgen signalling is known to play a crucial role in prostate cancer biology. While both surgical castration and hormonal antineoplastic agents have the shared therapeutic objective of inhibiting the production or action of DHT, metastatic prostate cancers often develop resistance [45, 46]. This suggests the acquisition of alternative mechanisms to either sustain AR signalling or bypass it entirely through intracellular or autocrine hormone production. More recently, bipolar androgen therapy (BAT) has sought to treat patients, resistant to hormone-blocking therapies, with an alternating high and low level of testosterone, causing a disruption to cell function [47]. As such, alterations to lysosomal biogenesis and dysregulated vesicular trafficking may be maintained or even enhanced by these new treatment modes. DHT is known to regulate TFEB expression [18], a master regulator of lysosomal biogenesis, which may explain the increased lysosome number following R1881 treatment. We hypothesise that androgen may enhance ER-lysosome contacts in the perinuclear region of cancer cells to promote lysosome fission; where ER-lysosome contacts have previously been observed to facilitate lysosome fission and axonal translocation [48], though we cannot rule out the effects of R1881 on tubulin acetylation that promotes motor binding and motility of kinesin1 and dynein [49, 50]. These observations have profound significance for androgen deprivation in patient management, as it can directly impact on lysosome-related processes and contribute to prostate cancer progression.

The dysregulated lysosome trafficking machinery identified in our study may provide potential biomarkers for prostate cancer diagnosis, prognosis, and therapeutic targeting. The altered expression of trafficking genes across different GPs and patient cohorts suggests their potential to risk stratify patients based on disease aggressiveness. Targeting lysosome vesicular trafficking machinery could represent a promising therapeutic strategy for prostate cancer. This is because modulating lysosome positioning and function can affect crucial cellular processes, such as autophagy, exocytosis, and secretion of factors involved in cancer pathogenesis [21]. Their crucial role in macromolecular degradation, antigen presentation, intracellular pathogen destruction, plasma membrane repair, exosome release, cell adhesion/migration, and apoptosis suggest that the functional status and spatial distribution of lysosomes are closely related to cancer cell proliferation, energy metabolism, invasion and metastasis, immune escape, and tumour-associated angiogenesis [21].

The modulation of EMT protein expression by lysosome trafficking machinery loss of function offers insight into how EMT may be regulated in prostate cancer cells to promote cancer cell migration and metastasis. The increased expression of EMT proteins and decrease in prostate cancer cell migration upon DYNC1I1 knockdown suggested that lysosome retrograde trafficking machinery is required to regulate EMT in prostate cancer cells. The increase in EMT proteins upon DYNC1I1 knockdown allude to dysregulated lysosomal function, as a decrease in intermediate light chains of dynein were shown to attenuate lysosome degradation [51]. The decrease in migration potential observed here was consistent with previous studies that have reported in ovarian cancer cells with increased E-cadherin having weaker invasiveness relative to cells with increased expression of N-cadherin [52]. The switch between epithelial to mesenchymal protein expression may therefore be important in the selective modulation of EMT protein expression involving alterations in the other lysosome trafficking machinery that dysregulate retrograde trafficking of lysosomes.

Extracellular vesicles (EVs) play an important role in intercellular communication and have shown potential for analysis in liquid biopsies from patients with prostate cancer; including specific biomarker expression in blood and urine samples from prostate cancer patients, which makes them a promising resource for early diagnosis and predicting the stages of cancer [53, 54]. Lysosomes play a crucial role in the biogenesis of EVs, including the packaging of specific cargo. Therefore, alterations in lysosome biogenesis could potentially influence the composition and function of EVs, affecting their role in disease pathogenesis and increasing their potential for analysis in liquid biopsies [53]. Understanding the implications of lysosome biogenesis on the properties and release of EVs may reveal pivotal roles in cellular communication and disease pathogenesis. The analysis of trafficking machinery in the context of EVs could provide valuable insights into disease progression and open new avenues for therapeutic intervention.

Our study had three limitations that should be considered when interpreting our results. First, we used meta-analysis of Oncomine data; and while Oncomine is a comprehensive resource, it may not fully represent all populations or capture the complete heterogeneity of prostate cancer biology. The studies within the Oncomine database may also be subject to sampling bias that could lead to over or underestimation of certain gene expression patterns, based on the tumour grade and overall percentage of cancer in the sample. Therefore, our results may not reflect the true expression levels of the genes of interest in tissue samples from patients with prostate cancer. Second, we used cell line models that may not fully reflect the complexity and diversity of human prostate cancer, especially as the prostate cancer cell lines used were derived from metastatic cancer patients. Third, we did not investigate the inheritance factors that may increase the risk of developing prostate cancer or indeed that could affect lysosome trafficking. Future studies should therefore validate our findings in larger and more diverse cohorts of patients, use in vitro organoid culture models and in vivo patient-derived xenografts models to mimic the tumour microenvironment, as well as exploring the genetic and epigenetic factors that may modulate lysosome trafficking in prostate cancer.

In conclusion, our study provides novel insights into the altered regulation of lysosomal vesicular trafficking machinery in prostate cancer and its potential implications for diagnosis, prognosis, and treatment. The disruptions in lysosome motility and altered expression of lysosome trafficking machinery genes highlight the importance of lysosome dynamics in prostate cancer biology. Our findings highlight a potential role of lysosome trafficking machinery in lethal CRPC that may be explored as an alternative avenue for disease therapy that, through restoring normal lysosomal vesicular trafficking could potentially inhibit cancer cell invasion and metastasis, enhance sensitivity to chemotherapy, and improve patient outcomes.

## Methods

### Cell culture

Human prostate cells PNT1a (#95012614), LNCaP (#89110211) and PC-3 (#90112714) were obtained from the European Collection of Authenticated Cell Cultures (ECACC) via CellBank Australia (Children’s Medical Research Institute, Westmead, NSW, Australia). All cell lines were authenticated by short tandem repeat (STR) profiling and were negative for mycoplasma contamination with frequent testing. PNT1a and LNCaP cells were maintained in RPMI 1640 medium (Gibco®, Thermo Fisher Scientific Australia Pty Ltd., VIC, Australia).PC-3 cells were maintained in Ham’s F12K medium (Gibco®). Culture media were supplemented with 10% (v/v) foetal bovine serum (FBS, Moregate Biotech Pty Ltd., QLD, Australia). Media were replenished every 2-3 d and cells sub-cultured at 70–80% confluency. Cell cultures were maintained at 37 °C with 5% CO_2_. Androgen treatment of LNCaP cells was performed 48 h after sub-culture; briefly, cells were allowed to adhere for 24 h before culture medium was replaced with RPMI supplemented with charcoal stripped FBS (CS-FBS). After 24 h, 1, 10 or 100 nM synthetic dihydrotestosterone (DHT) (R1881; Sigma-Aldrich Pty Ltd., NSW, Australia), or vehicle (0.01% v/v ethanol), was added. Cells were incubated with vehicle or R1881 for 48 h. For all experiments, cells were seeded at densities to reach 80% confluency after 5 d (PNT1a; 3× 10^4^ cells/cm^2^, LNCaP; 3 × 10^4^ cells/cm^2^, PC-3; 1.3 × 10^4^ cells/cm^2^).

### Immunoblotting

Cell lysates were prepared from cultured cells, washed with PBS and treated with RIPA lysis buffer (Merck Millipore) supplemented with Halt™ protease and phosphatase inhibitor cocktail (Thermo Scientific). After centrifugation, the supernatant was transferred to fresh Eppendorf tubes and stored at −30 °C. Protein amount was determined using Micro BCA Protein Assay Kit (Thermo Scientific) as per manufacturer’s instructions.

Ten micrograms of total cell protein were heat-denatured, electrophoresed and transferred to a PVDF membrane (Thermo Fisher). The membranes were blocked, incubated with primary antibody overnight, washed and then incubated with the appropriate fluorescent conjugated secondary antibody diluted 1:15 000 in block (Table 1). Membranes were imaged using an Odyssey® Imaging System (Li-COR Biosciences, USA). Signal intensity was quantified and normalised to GAPDH or total protein using Image Studio (v5.3; Li-COR). Statistical analysis was performed using RStudio (2022.x; RStudio, Boston, USA) with linear mixed effects modelling (LME). Differences between groups were tested at a 95% confidence level.

**Table 1:** Antibody reagents

Protein target	Cat#	Host/Clonality	Western/BLOCK	IHC	IF
GAPDH (GA1R)	MA5-15738	MOUSE	1:6000, 5% BSA		
GAPDH DyLight™ 680	MA5-15738-D680	MOUSE	1:6000, 3% BSA		
GAPDH (14C10)	#2118	RABBIT MONOCLONAL	1:2000, 5% BSA		
Rabbit IgG 647	A31573	Donkey			1:1000
Mouse IgG IRDye® 680RD	925-68070	Goat	1:15 000, 3% BSA		
Rabbit IgG IRDye® 800CW	925-32211	Goat	1:15 000, 3% BSA		
LAMP1	AB24170	RABBIT POLYCLONAL			0.25 µg/mL
ARF6	PA5-44191	RABBIT POLYCLONAL	0.2 µg/mL, 3% BSA	1.25 µg/mL	
DCTN1	AB11806	GOAT POLYCLONAL	1:1000, 3% BSA	1.25 µg/mL	
DYNC1I1	AB23905	MOUSE MONOCLONAL	1:1000, 5% MILK	0.5 µg/mL	
DYNC1H1	GTX101452	RABBIT POLYCLONAL	1:1000, 3% BSA	8.9 µg/mL	
JIP3	AB196761	RABBIT POLYCLONAL	2 µg/mL, 3% BSA	0.5 µg/mL	
	AB209337	RABBIT POLYCLONAL	2 µg/mL, 3% BSA		
JIP4	D72F4(5519)	RABBIT MONOCLONAL	1:1000, 3% BSA	10 µg/mL	
KIF5A	AB154414	RABBIT POLYCLONAL	2 µg/ml, 3% BSA	1:200	
KIF5B	AB167429	RABBIT MONOCLONAL	0.141 µg/mL, 3% BSA	1:200	
	AB25715	RABBIT POLYCLONAL	0.141 µg/mL, 3% BSA		
KIF5C	AB193352	RABBIT MONOCLONAL	1.67 µg/mL, 3% BSA	1:200	
KLC1	AB174273	RABBIT MONOCLONAL	0.321 µg/mL, 3% BSA	1:200	
KLC2	AB254848	RABBIT POLYCLONAL	0.6 µg/mL, 3% BSA	1:200	
PLEKHM1	HPA025018	RABBIT POLYCLONAL	1:3000, 3% BSA	1:200	
PLEKHM2	SAB35000137	RABBIT POLYCLONAL	1:3000, 5% Milk	1:200	
ARL8A	HPA045924	RABBIT POLYCLONAL	1:500, 1:1 Intercept	1:100	
ARL8B	CST.56085	RABBIT POLYCLONAL	1:1000, 3% BSA	1:100	
E-Cadherin	MAB18381	MOUSE MONOCLONAL	1:250, 5% Milk		
EpCAM	AB213500	RABBIT MONOCLONAL	1:1000, 5% Milk		
Vimentin	AB16700	RABBIT MONOCLONAL	1:500, 5% Milk		
N-Cadherin	#13116	RABBIT MONOCLONAL	1:1000, 5% Milk		

### qPCR

Relative gene expression of non-malignant and prostate cancer cell lines was assessed via qPCR. RNA was isolated from cell pellets, quantified, and 1 µg of RNA was reverse transcribed. qPCR was performed on a 1:10 dilution of 2 µg cDNA using TaqMan or PowerUp SyBr green (A25741, Thermo Fisher) master mix and assay (Tables 2 and 3) on a Viia7 Real-Time PCR System or QuantStudio™ 7 Pro instrument (Thermo Fisher). Three biological replicates were analysed. ACTB, HPRT1, and GAPDH were used as endogenous controls for normalisation using the ΔΔCT method. Differences between cell lines were tested for significance using Kruskal-Wallis nonparametric tests.

**Table 2:** TaqMan assays

Taqman Assay ID	Gene target
Hs99999903_m1	ACTB
Hs01066735_g1	ARF6
Hs00373395_m1	ARL8A
Hs04969687_g1	ARL8B
Hs00896389_g1	DCTN1
Hs00292485_m1	DNAH5
Hs00322286_m1	DYNC1H1
Hs00189392_m1	DYNC1I1
Hs99999905_m1	GAPDH
Hs99999909_m1	HPRT1
Hs00192120_m1	KIF5A
Hs01037194_m1	KIF5B
Hs00189672_m1	KIF5C
Hs00194316_m1	KLC1
Hs03988192_m1	KLC2
Hs01049862_m1	MAPK8IP3
Hs00292741_m1	PIP4P1
Hs04194441_g1	PLEKHM2
Hs00187715_m1	SPAG9

**Table 3:** Sequences of primers used in qPCR

Gene target	Primer Sequence (5’ - > 3’)	
	Forward	Reverse
DYNC1H1	GCTAAGAGCTGTTATCGTCAGG	GCCACTTTCATATCTTGGGGTTC
DYNC1I1	AAAGCTGAGCTAGAGCGCAAA	GTCCTGAACGGGTTCTTTCTTC
KIF5B	AGATCCTGCGGAACACTATTCA	GCGGTTGCTGGTTTATCATTGG
KIF5C	ATGTCTTCGACAGAGTGCTACC	ACGCAAAAATCGTCCCGTTAT
MAPK8IP3	TGGAGCACTACGAGTTCCAGA	CTGGTGCAGGGCATTGTACT
PLEKHM1	AGCACCTGACGTTTGTCAACA	TGGCAGACTTGTAGGAGAGTTC
PLEKHM2	GGTGCTCGTGGTGCATTTTAC	CAGGTAGCTCTCCAAGGAGTT
GAPDH	ACCCAGAAGACTGTGGATGG	CAGTGAGCTTCCCGTTCAG

### RNASeq

Sequencing was performed on an Illumina Nextseq sequencer, producing an average of 28 million 74 nucleotide single-end reads per RNA sample (range 21–33 million reads). Raw reads were analysed using FastQC (version v0.11.9) to assess quality and adaptor contamination [https://www.bioinformatics.babraham.ac.uk/projects/fastqc/]. Adaptors were trimmed using cutadapt (version 1.18) [55] with the following parameters: -a AGATCGGAAGAGCACACGTCTGAACTCCAGTCA –minimum-length 18 –error-rate 0.2 –overlap 5. Trimmed reads were aligned to the hg38 reference genome using STAR (version 2.7.10a) [56]. PCR duplicates were removed using UMI-tools (version 1.1.2) [57], before deduplicated reads were re-mapped using STAR, producing a gene counts matrix.

Differential gene expression analysis of these gene counts was performed in RStudio v2022.07.1 + 554 (RStudio, Boston, USA) with R v4.2.2 (R Core Team, Vienna, Austria) using the Bioconductor package edgeR (version 3.38.4) [58]. Lowly expressed genes were first filtered by requiring greater than three counts per million in at least three libraries. Library size normalisation factors were calculated using the calcNormFactors function, enabling accurate comparisons of gene expression between samples. Data were explored using a multidimensional scaling (MDS) plot generated using plotMDS, allowing assessment of replicate sample clustering and treatment effects. A negative binomial generalised log-linear model was fit to the read counts for each gene using glmFit, utilising a design matrix including a term describing experimental batch. A likelihood ratio test was performed using glmLRT to test the difference in gene expression between treatments.

### Oncomine analysis

To identify trafficking machinery genes that may be altered in prostate cancer, differential gene expression analysis was conducted on prostate cancer cohorts using the Oncomine™ database [59]. This database comprised microarray data from 16 peer-reviewed publications which together spans 851 clinical samples across 15 cohorts of patients with prostate cancer. Differentially expressed genes were further filtered at a statistical threshold of *P* < 0.05 and fold change > 1.5. Prostate cohorts explored included: Arredouani Prostate, Grasso Prostate, Holzbeierlein Prostate, LaTulippe Prostate, Lapointe Prostate, Liu Prostate, Luo Prostate 2, Singh Prostate, Taylor Prostate 3, Tomlins Prostate, Vanaja Prostate, Varambally Prostate, Wallace Prostate, Welsh Prostate and Yu Prostate [59].

### TCGA-PRAD analysis

The Cancer Genome Atlas Prostate Adenocarcinoma (TCGA-PRAD) [20] RNASeq dataset was downloaded, processed, and analysed using the TCGA biolinks R package [60] as per the package vignette. Briefly, RNASeq data was downloaded, and lowly expressed genes were filtered at the default quantile threshold of 0.25. Differential gene expression analysis between benign and the Gleason grades of prostate cancer was then defined using the edgeR pipeline within TCGA biolinks to fit negative binomial generalised log-linear models to the read counts and to apply false discovery rate (FDR) correction to the statistical analysis. A gene expression data table was extracted and filtered for the trafficking machinery genes of interest. The fold-changes of differentially expressed genes were plotted on a heatmap using pHeatmap (v 1.0.12).

For gene correlation analysis, the log counts per million (cpm) for each gene in each of the prostate Gleason grades were back transformed and the Pearson correlation coefficient between each gene was calculated in RStudio. The gene correlation coefficients for Gleason grade 3 were clustered in a heatmap based on similar correlation patterns and the order of clustered genes preserved in benign, Gleason pattern / grade 4 and Gleason pattern / grade 5 analyses.

### K-means clustering of Glinsky and APCB cohort gene expression

Patients were classified into two groups based on “higher” and “lower” mRNA expression using K-means clustering, as previously described ([4]; Fig. 3). Briefly, K-means clustering was performed using Cluster 3.0 [61] to determine high and low gene expression groups. These groups were evaluated using Kaplan-Meier survival curves with differences determined using the Log-Rank (Mantel-Cox) test using GraphPad Prism (v9.x for Windows; GraphPad Software, MA, USA). Affymetrix probes selected from Glinsky cohort included *DCTN1* (probe 201082_s_at), *PLEKHM1* (216200_at), *ARL8B* (217852_s_at) and *DYNC1I1* (205348_s_at).

### Cell migration

For knockdown experiments, siRNA SMARTpool ON-TARGETplus (Dharmacon Inc., Lafayette, USA; Table 4) was used for reverse transfection. 25 nM of scrambled or target siRNA were diluted in Opti-MEM media (Gibco®) and mixed with Lipofectamine™ RNAiMAX Transfection Reagent (Thermo Fisher) suspended in Opti-MEM media. The mixture was added to freshly seeded PNT1a, LNCaP, and PC 3 cells for migration assays and lysate extraction to validate transfection efficiency. For cell migration, cells were transfected for 72 h with scrambled or target siRNA. A wound was created using an Incucyte® Wound Maker Tool (Sartorius, Gräfelfing, Germany) or a 20 µL micropipette tip. The wound healing process was monitored for 24 h on the CellDiscoverer 7 (Carl Zeiss Microscopy GmbH, Munich, Germany) using a brightfield 5× objective lens. Wounded regions of interest were analysed using ZEN software. Automated image analysis was conducted using skimage image processing in python (v 3.9.12) to calculate the wounded area. The mean percentage of wound closure was calculated, and LME models were applied using RStudio to test if the rate of wound closure in siRNA treated cells were significantly different to the controls.

**Table 4:** SiRNA reagents

Target	Cat#
ARL8A	L-016577-01-0005
ARL8B	L-020294-01-0005
PLEKHM1	L-023203-01-0005
PLEKHM2	L-022168-01-0005
KLC1	L-019482-00-0005
KLC2	L-014218-00-0005
DYNC1H1	L-006828-00-0005
DYNC1I1	L-019799-00-0005
ARF6	L-004008-00-0005
KIF5A	L-008559-00-0005
KIF5B	L-008867-00-0005
KIF5C	L-019811-01-0005
JIP3	L-003596-00-0005
JIP4	L-017462-00-0005
DCTN1	L-012874-00-0005
GAPDH	D-001830-10-05
Non-targeting pool (scrambled)	D-001810-10-05

### Lysosome tracking

For lysosome tracking, PNT1a, LNCaP and PC-3 cells (1.4 × 10^4^ cells/cm2) were seeded for 24 h in their respective media in a chambered coverslip (µ-Slide 8 Well; ibidi GmbH, Gräfelfing, Germany). LAMP1-GFP plasmid (detailed in materials) was prepared at 0.5 µg/cm^2^ with Lipofectamine™ 2000 Transfection Reagent (Thermo Fisher) as per manufacturers’ instructions. The mixture was added to cells and after 4 h incubation, media was aspirated and fresh medium containing 10% FBS was added. For lysosome trafficking speed in R1881-treated LNCaP cells, LNCaP cells were first cultured in RPMI media with 10% FBS prior to aspiration and incubation for a further 24 h with RPMI + 10% charcoal-stripped FBS (CS-FBS). Media was replaced with LAMP1 plasmid in Opti-MEM for 4 h, then aspirated and replaced with RPMI with CS-FBS containing 0, 10, or 100 nM R1881, diluted in ethanol. At 24 h post-transfection, live-cell imaging was performed as detailed below.

To determine lysosome location and cell morphology, over-expression plasmids of trafficking machinery (detailed in materials) were prepared and introduced into cells with Lipofectamine™ 3000 as per manufacturer’s instructions on coverslips in 24 well plates. 24 h post-transfection, cells were fixed and immunolabelled with LAMP1 as described below. To determine lysosome location from R1881 treatment, LNCaP cells were cultured on coverslips in 24 well plates for 24 h in RPMI and 10% FBS, media gently aspirated and RPMI + 10% CS-FBS added. Cells were incubated for 24 h, half the media (200 µL) aspirated and 200 µL fresh media + 10% CS-FCS containing 0, 2, 20 or 200 nM R1881 added to create a final concentration of 0, 1, 10 and 100 nM R1881.

### Immunolabelling

Immunolabelling was achieved through cell fixation with 4% PFA in PBS containing 4% w/v sucrose for 10 min. Cells were washed, blocked and permeabilised (5% (w/v) BSA and 0.05% (w/v) saponin). Cells were probed with LAMP1 antibody at 37 ^o^C for 20 min. Cells were washed and incubated with anti-rabbit AlexaFluor conjugated secondary antibody (1:1000, #A31573, Thermo Fisher) and Hoechst (1:1000, #33342; Thermo Fisher). Coverslips were mounted on slides using Prolong Glass Antifade (#P36980; Thermo Fisher).

### Microscopy

All fluorescence microscopy was performed on the Nikon A1+ confocal microscope (Nikon, Tokyo, Japan) equipped with a LU-N4/LU-N4S 4-laser unit using a Plan Apo λ 60× oil-immersion objective lens with NIS Elements software (v4.5, Nikon). Each experiment was repeated three times, with ten cells captured per replicate. For live cell imaging of lysosome traffic, LAMP1-transfected cells were incubated with the Uno-combined-controller, CO_2_ microscope electric top stage incubation system (Okolab, Pozzuoli, NA, Italy). Imaging was performed using resonant scanner at 512-pixel resolution, piezo z-stage, 2× line averaging with 3× zoom (0.14 μm/px) and 18 z-steps of 0.4 μm were imaged, with 100 3D frames obtained ( ~ 2.5 min per cell). Lysosome tracking was performed using Imaris software (v9.7.0, Oxford Instruments, Oxford, England).

To visualise lysosome distribution, fixed R1881-treated LNCaP cells were captured as above with resonant scanner, 4× line averaging, and z-stacks of 0.175 µm step depth. Distance to the nucleus surface was determined using spots analysis of vesicles with Imaris. To visualise lysosomes, GFP-fluorescent cells were selected using epifluorescence, and images acquired at 3× scanner zoom, at 512 px with z-stacks of 0.25 µm step depth. Cell analysis was performed in Imaris to determine the shortest distance between lysosome centre to nucleus surface (Supplementary video 2) and the morphology of the cell.

### Detection of lysosome trafficking speed and distance

Lysosome speed was determined using spots tracking analysis in Imaris. LAMP1-GFP vesicles were determined using spots detection algorithm; estimated XY diameter 0.4 µm, estimated Z diameter 1.20 µm, background subtraction = true. Spots were filtered by “Quality” above automatic threshold. Tracking was performed using autoregressive motion algorithm with MaxDistance 2.5 µm and MaxGapSize 0, and filtered for duration greater than 6 sec. Generalised linear models were fitted to the data with Gaussian distributions using the glmmTMB R package [62], to determine statistical differences in lysosome mean-track-speed.

Lysosome distance from cell nuclei were determined through spots analysis in Imaris from cell detection algorithm; vesicle estimated diameter 0.4 µm, vesicle background subtraction true. Vesicles were selected from an intensity level greater than 1350. The kernel density estimates of LAMP1 positive vesicle distribution from the nucleus surface were calculated with bandwidth and graphed using the ggplot2 package in RStudio. Generalised linear models were fitted to the data with Tweedie distributions using the glmmTMB R package [62], to determine if differences in LAMP1 distributions in control and cDNA or siRNA treated cells were statistically significant.

### Measurement of cell morphology and lysosome number

The ellipticity (oblate), width and length of cells were calculated in cells over-expressing trafficking machinery using the cellular boundaries from the corresponding reporters of transfection efficiency (mCherry or GFP; Table 5), as described above. Lysosome number was quantified by enumerating the spots with LAMP1 positive labelling. Linear mixed effects (LME) models were fitted to determine if differences observed in cell morphology and lysosome number of control and cDNA treated cells were statistically significant.

**Table 5:** Plasmid constructs

Protein	Accession number	Tag location
KIF5C	NP_004513_1	C-terminal
KLC1	NP_005543_2	N terminal
KLC2	NP_001128247_1	N terminal
JIP3	NP_055948_2	N terminal
JIP4	NP_001124000_1	N terminal
ARL8B	NP_060654_1	N terminal (mCherry)
GFP	MZ420392.1	N.A.
mCherry	MZ027319.1	N.A

### Immunohistochemistry

Formalin fixed, paraffin embedded (FFPE) prostate tissue for immunohistochemical (IHC) assessment was obtained from Australia Prostate Cancer Bioresource (APCB; Adelaide, Australia). Tissue was sourced from male patients with primary prostatic adenocarcinoma (the median age at the time of surgery was 62 years, ranging from 47 to 71 years) diagnosed between 1987 and 1990. Approval specific to this study was obtained from the Human Research Ethics Committee of the University of South Australia (Application IDs: 201907 and 36070).

Serial sections (2 µm) of tissue were cut and stained with routine haematoxylin (Ehrlich’s) and eosin (H&E) or labelled by IHC as previously described [63]. Briefly, sections were deparaffinised in xylene and rehydrated with graded ethanol solutions. Heat-induced epitope retrieval was performed in Tris-EDTA Buffer (10 mM Tris Base, 1 mM EDTA, 0.05% Tween®-20, pH 9.0). Detection of immunolabelling was performed using the DAKO EnVision+ kit (Dako Australia Pty Ltd., NSW, Australia) as per manufacturer’s instructions. Tissue sections were counterstained with Ehrlich’s haematoxylin. Sections were cleared in xylene and mounted in DPX mounting media (Merck Millipore Pty Ltd., VIC, Australia). Slides were imaged in brightfield with a ZEISS Axio Scan Z.1 slide scanner with a Plan-Achromat 20× (0.8 NA) objective (Carl Zeiss Microscopy GmbH, Munich, Germany). H&E sections and IHC sections with antibody labelling were assessed and representative regions of glands were annotated for benign, Gleason pattern 3, Gleason pattern 4, and Gleason pattern 5 glands. A modified H-score method was used as previously described [64] to measure the degree of DAB staining intensity, as a measure of expression, in each Gleason pattern at a range of 0 to 3.

### Materials

#### Plasmids

Transfected into cells at an equivalence of 0.25 µg/cm^2^, Plasmids were created by GeneArt (Thermo Fisher; Table 5), with GFP or mCherry tags (GenBank ID MZ420392.1, MZ027319.1). PLEKHM2/ SKIP-GFP and LAMP1-GFP was provided as a gift from Prof J Bonifacino.

## Supplementary information

Supplemental Materials

Supplementary Dataset 1

Supplementary Videos 1

Supplementary Videos 2

## Data Availability

Data is available from the corresponding author BDN. Publicly archived datasets analysed include TCGA-PRAD. RNA sequencing data from R1881-treated LNCaP cells are available on the Gene Expression Omnibus (GEO) – NCBI – NIH repository under the accession number GSE220618.
