# Supplementary material for: Altered expression of vesicular trafficking machinery in prostate cancer affects lysosomal dynamics and provides insight into the underlying biology and disease progression
Source: Br J Cancer. 2024 Aug 31;131(8):1263–78. doi: 10.1038/s41416-024-02829-x (PMC11473802; doi:10.1038/s41416-024-02829-x)
Supplement: Supplementary file 1 — Supplemental Materials [file 41416_2024_2829_MOESM1_ESM.docx]

**Altered expression of vesicular trafficking machinery in prostate cancer affects lysosomal dynamics and provides insight into the underlying biology and disease progression**

Bukuru D Nturubika^1*^, Carlos M Guardia^2^, David C Gershlick^3^, Jessica M Logan^1^, Carmela Martini^1^, Jessica K Heatlie^1^, Joanna Lazniewska^1^, Courtney Moore^1^, Giang T Lam^1^, Ka L Li^1^, Ben S-Y Ung^4^, Robert D Brooks^1^, Shane M Hickey^1^, Andrew G. Bert^5^, Philip A. Gregory^5^, Lisa M Butler^6,7^, John J O’Leary^8^, Douglas A Brooks^1*#^, Ian R D Johnson^1 #^

**Supplementary Figure 1**


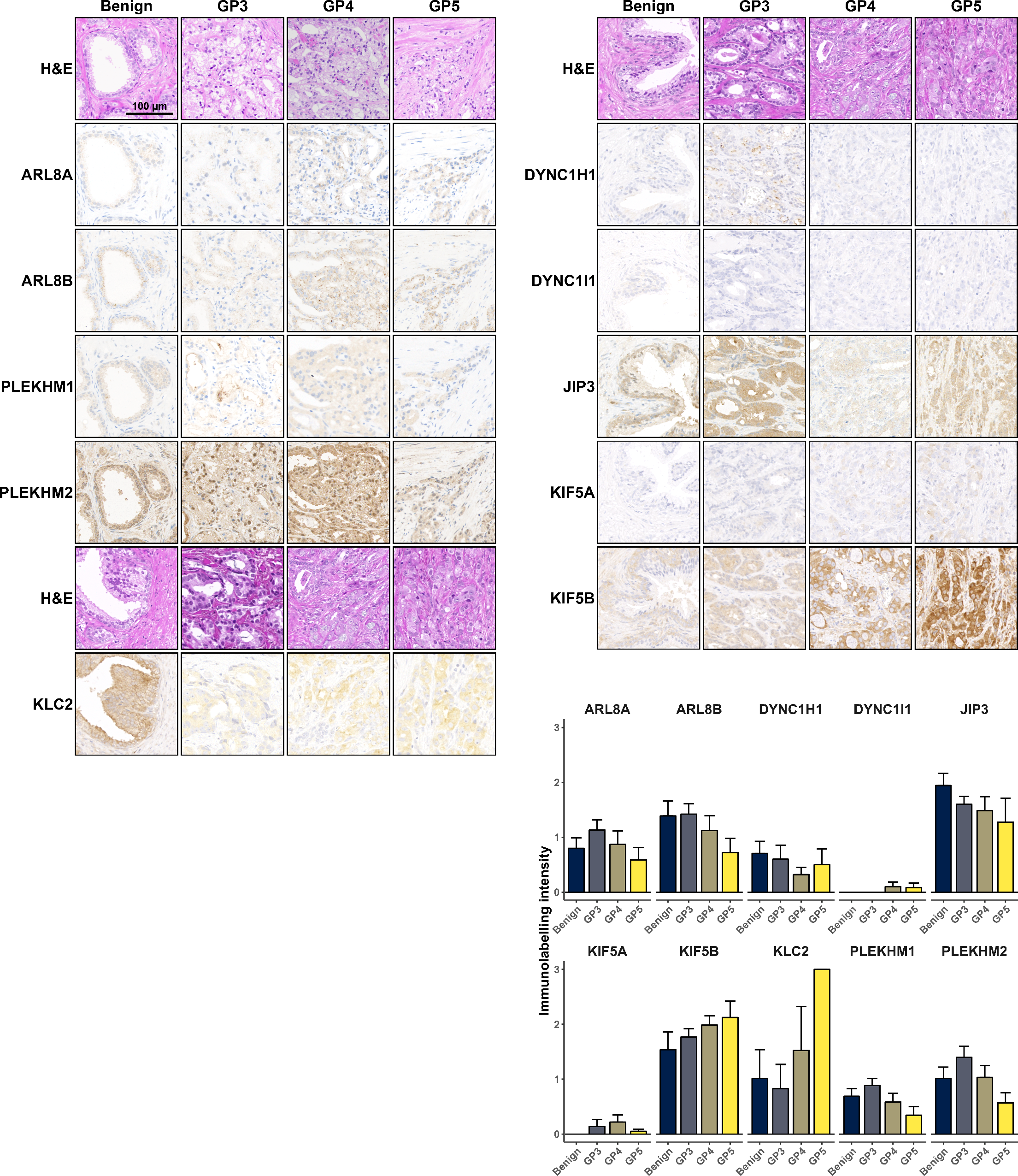


Supplementary Figure 1.

Detection and quantification by modified H-score of immunolabelling of lysosome related trafficking machinery in prostate tissue. Scale bar, 100 µm.

***Supplementary Figure 2***


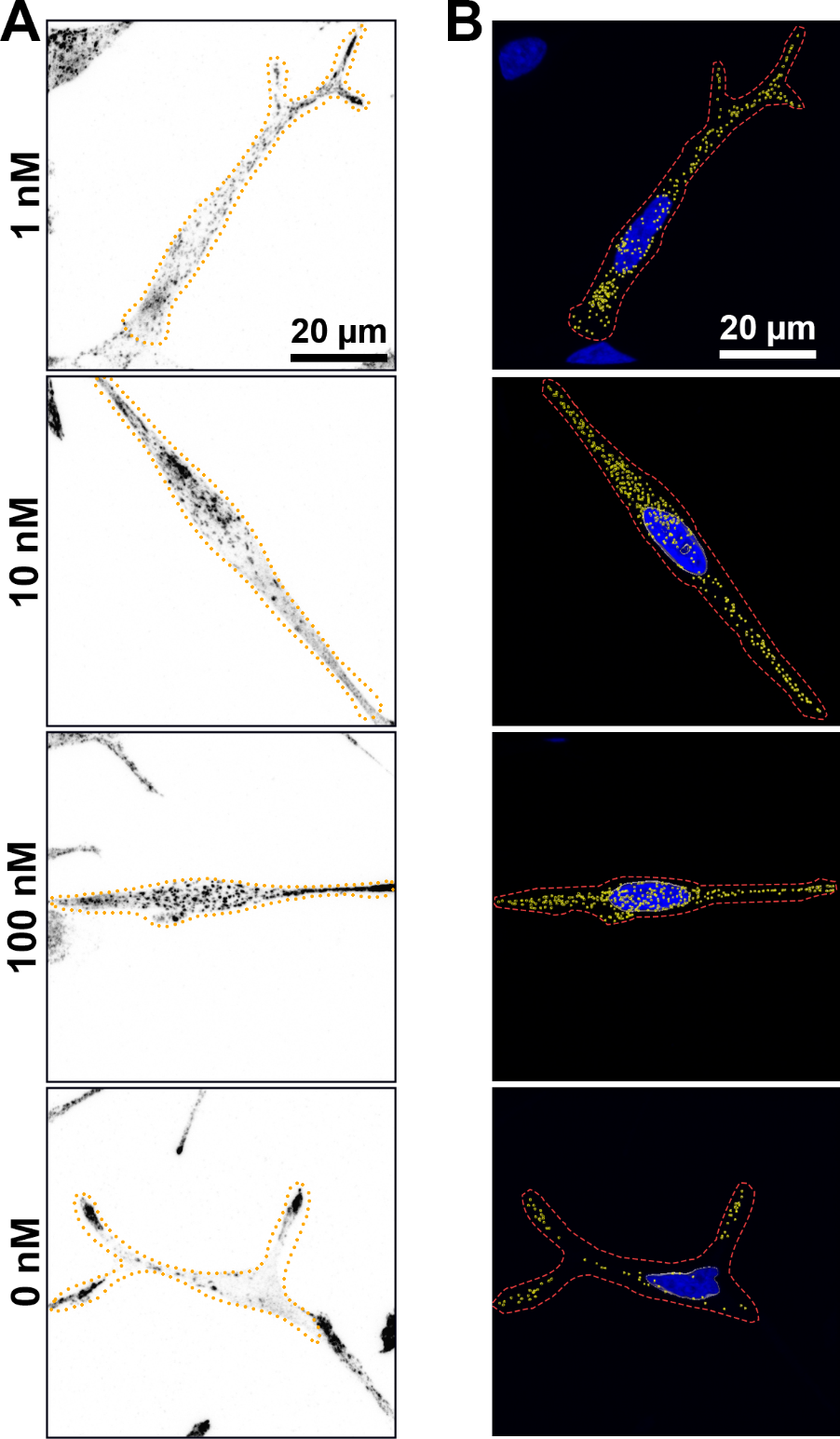


**Supplementary Figure 2**

Hormone-sensitive cells exhibit a spatiotemporal response of lysosomes to 48 h R1881 treatment. **A**, 2D Representative maximum-intensity projections of micrographs and **B,** 3D spot detection of lysosome subcellular location from 0, 1, 10 and 100 nM R1881 treatment of LNCaP cells. Scale bar, 20 µm.

***Supplementary Figure 3***


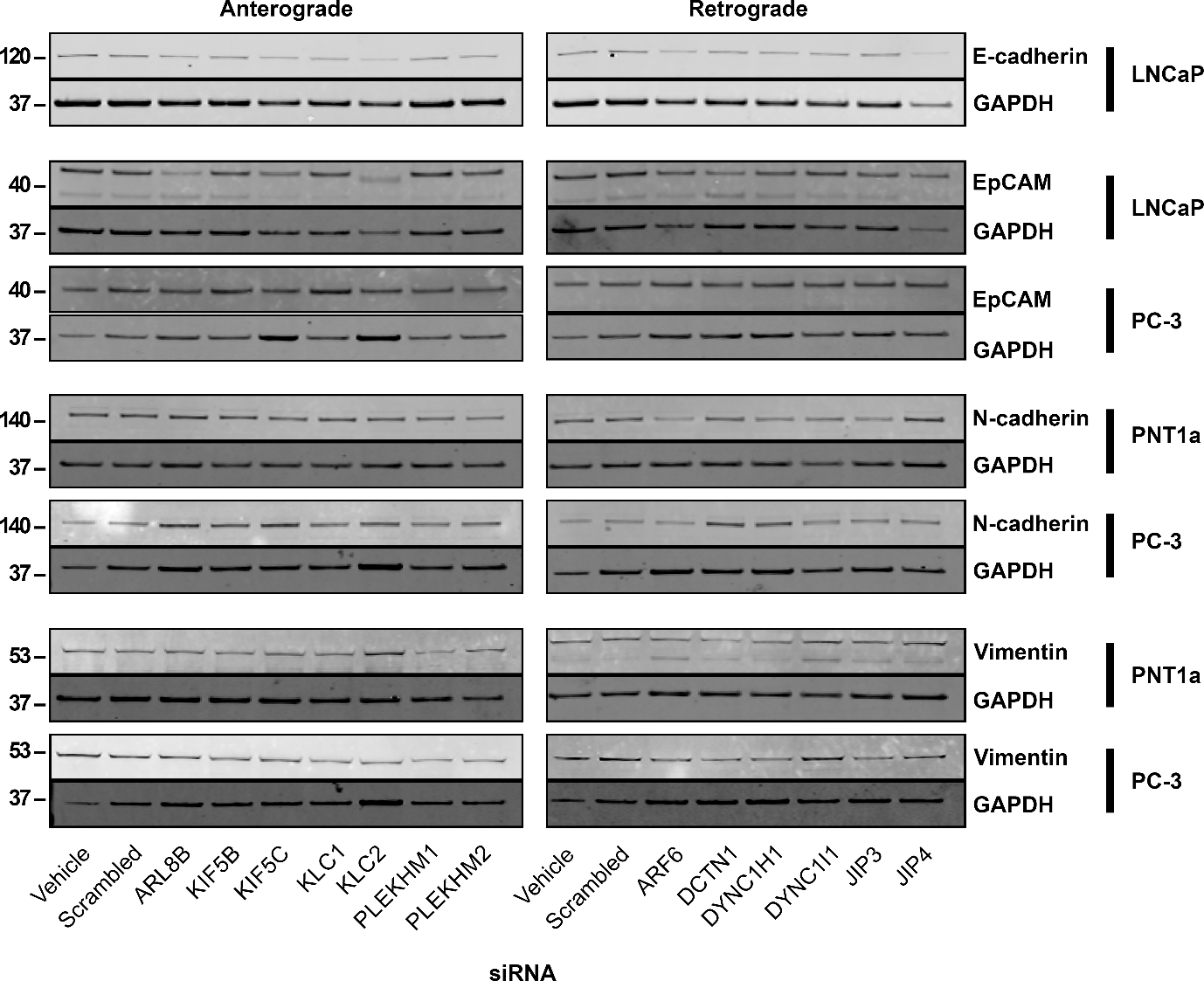


**Supplementary Figure 3**

Representative Western blots from three biological replicates of siRNA experiments targeting lysosome trafficking machinery in PNT1a, LNCaP and PC-3 cells. Western blot images have been cropped from full-length gels and membranes immunolabelled for each of the epithelial to mesenchymal proteins.

**Supplementary Figure 4**

**
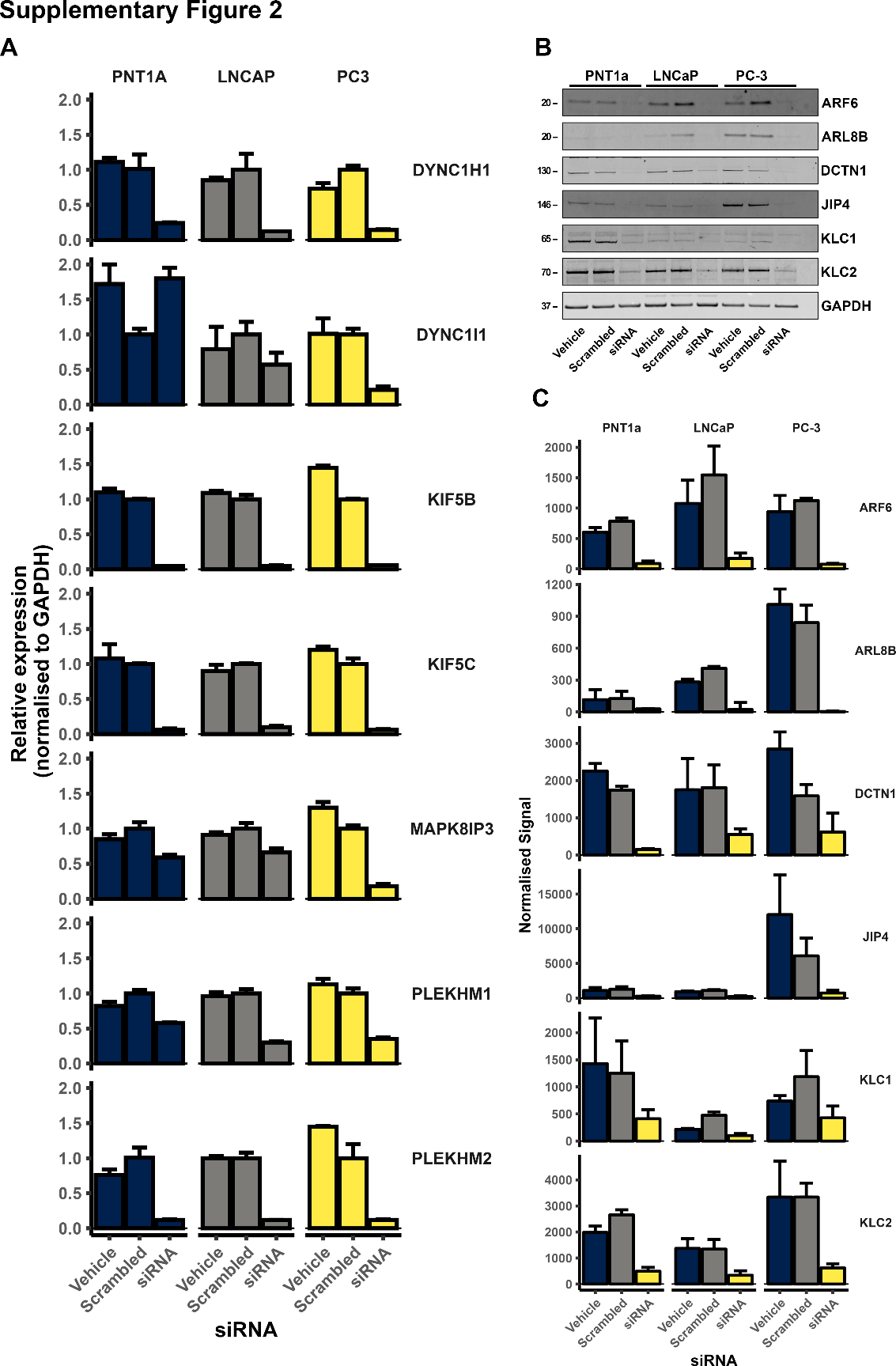
**

**Supplementary Figure 4**

Validation of transfection efficiency in PNT1a, LNCaP and PC-3 cells following 72 hour siRNA knockdown of lysosome trafficking machinery. **A**, qPCR quantification of mRNA expression of trafficking machinery relative to scrambled, normalised to GAPDH. Error bars indicate the standard deviation (SD). **B& C**, Representative Western blots and quantification of siRNA efficacy normalised to GAPDH in PNT1a, LNCaP and PC-3 cells. Western blot images have been cropped from full-length gels and membranes labelled by each antibody. Error bars indicate the standard error of the mean (SEM).

### Supplementary Table 1: Fold change mRNA expression in LNCaP and PC‑3 cells prostate cancer cells compared to non-malignant PNT1a cells. Related to Figure 1.

| mRNA | PNT1a vs. | Fold Change | *P* Value | Summary |
| --- | --- | --- | --- | --- |
| *ARL8A* | LNCaP | 1.08 | 0.914 | ns |
|  | PC‑3 | 0.85 | 0.1623 | ns |
| *ARL8B* | LNCaP | 0.98 | 0.3885 | ns |
|  | PC‑3 | 2.53 | <0.0001 | **** |
| *KIF5A* | LNCaP | 0.05 | <0.0001 | **** |
|  | PC‑3 | 0.01 | <0.0001 | **** |
| *KIF5B* | LNCaP | 0.65 | <0.0001 | **** |
|  | PC‑3 | 0.7 | 0.005 | ** |
| *KIF5C* | LNCaP | 4.74 | <0.0001 | **** |
|  | PC‑3 | 10.74 | <0.0001 | **** |
| *KLC1* | LNCaP | 0.33 | <0.0001 | **** |
|  | PC‑3 | 1.24 | 0.1074 | ns |
| *KLC2* | LNCaP | 1.48 | 0.3885 | ns |
|  | PC‑3 | 3.56 | <0.0001 | **** |
| *PLEKHM1* | LNCaP | 0.85 | 0.0124 | * |
|  | PC‑3 | 3.04 | <0.0001 | **** |
| *PLEKHM2* (SKIP) | LNCaP | 1.57 | 0.2583 | ns |
|  | PC‑3 | 3.76 | <0.0001 | **** |
| *ARF6* | LNCaP | 1.1 | 0.4833 | ns |
|  | PC‑3 | 1.64 | 0.0005 | *** |
| *DCTN1* | LNCaP | 0.39 | <0.0001 | **** |
|  | PC‑3 | 1.25 | 0.0963 | ns |
| *DYNC1H1* | LNCaP | 0.82 | <0.0001 | **** |
|  | PC‑3 | 2.4 | <0.0001 | **** |
| *DYNC1I1* | LNCaP | 36.6 | <0.0001 | **** |
|  | PC‑3 | 87.68 | <0.0001 | **** |
| *MAPK8IP3* (JIP3) | LNCaP | 0.57 | <0.0001 | **** |
|  | PC‑3 | 0.78 | 0.0478 | * |
| *SPAG9* (JIP4) | LNCaP | 0.45 | <0.0001 | **** |
|  | PC‑3 | 4.74 | <0.0001 | **** |

### Supplementary Table 2: TCGA-PRAD mRNA fold change in cancer versus benign tissue.

| mRNA | Fold change | *P* value |
| --- | --- | --- |
| *ARF6* | 1.32 | 3.65E−09 |
| *ARL8A* | 1.13 | 1.08E−03 |
| *ARL8B* | −1.16 | 1.54E−04 |
| *DYNC1I1* | −2.28 | 2.73E−19 |
| *DCTN1* | −1.11 | 4.88E−02 |
| *KIF5A* | −1.67 | 2.07E−04 |
| *KIF5B* | −1.13 | 2.23E−02 |
| *KIF5C* | 1.25 | 8.59E−03 |
| *KLC1* | 1.32 | 4.94E−06 |
| *KLC2* | 1.27 | 2.10E−07 |
| *MAPK8IP3* | 1.38 | 4.08E−04 |
| *PIP4P1* | 1.09 | 1.81E−02 |
| *PLEKHM1* | −1.24 | 8.81E−07 |
| *PLEKHM2* | 1.08 | 1.90E−02 |
| *SPAG9* | −1.28 | 6.75E−06 |

### Supplementary Table 3: Meta analysis of select lysosome trafficking machinery in patient cohorts with prostate cancer.

| **Gene** | **PCa vs. Normal** | **Fold change** | ***P* value** | **Probe/reporter** | **Cohort** |
| --- | --- | --- | --- | --- | --- |
| ***ARF6*** | Up | 1.90 | 2.46E−04 | IMAGE:360885 | Tomlins |
|  | Up | 2.12 | 2.53E−04 | 37984_s_at | Singh |
|  | Up | 1.70 | 5.73E−06 | 37984_s_at | Welsh |
|  | Up | 1.56 | 0.006 | RC_AA291260_at | Luo |
|  | Up | 1.51 | 9.30E−04 | 37984_s_at | Yu |
| ***DCTN1*** | Up | 1.58 | 4.14E−04 | IMAGE:877613 | Tomlins |
|  | Up | 1.86 | 0.002 | 201082_s_at | Wallace |
|  | Down | −1.32 | 2.10E−04 | 36158_1at | Yu |
|  | Down | −1.22 | 0.007 | 201082_s_at | Arredouani |
|  | Down | −1.13 | 1.83E−06 | 16899 | Taylor |
| ***DYNC1I1*** | Down | −3.49 | 1.43E−05 | 40318_at | Welsh |
|  | Down | −2.35 | 0.001 | 40318_at | LaTulippe |
|  | Down | −1.57 | 0.004 | IMAGE:43865 | Tomlins |
|  | Down | −1.83 | 0.003 | 205348_s_at | Arredouani |
|  | Down | −1.71 | 0.002 | 40319_at | Singh |
|  | Down | −1.70 | 8.26E−08 | IMAGE:43865 | Lapointe |
| ***DYNC1H1*** | Up | 1.41 | 4.14E−04 | 229115_at | Vanaja |
|  | Up | 1.31 | 0.002 | 1556831_at | Arredouani |
|  | Down | −1.35 | 7.66E−04 | 229115_at | Varambally |
|  | Down | −2.50 | 0.032 | RC_T86293_at | Luo |
| ***SPAG9*** | Up | 1.99 | 1.66E−04 | IMAGE:278556 | Tomlins |
|  | Down | −3.23 | 0.038 | AA258972_at | Luo |
|  | Down | −1.77 | 0.008 | 212468_at | Arredouani |
|  | Down | −1.62 | 1.87E−05 | 212468_at | Wallace |
|  | Down | −1.42 | 7.19E−08 | IMAGE:247614 | Lapointe |
| ***ARL8A*** | Up | 1.60 | 0.003 | IMAGE: 592525 | Tomlins |
| ***ARL8B*** | Down | −1.55 | 7.31E−04 | IMAGE: 39677 | Tomlins |
| ***KIF5A*** | Up | 7.38 | 0.041 | U06698_at | Magee |
|  | Up | 3.71 | 0.01 | 205318_at | Varambally |
|  | Down | −1.59 | 0.04 | IMAGE:839641 | Tomlins |
|  | Down | −1.64 | 0.008 | 35880_at | Singh |
| ***KIF5C*** | Up | 5.97 | 7.70E−08 | IMAGE:49630 | Tomlins |
|  | Up | 2.14 | 3.31E−11 | IMAGE:49630 | Lapointe |
|  | Up | 2.13 | 7.07E−05 | 203129_s_at | Vanaja |
|  | Up | 1.87 | 7.94E−04 | 35778_at | Welsh |
| ***KLC1*** | Down | −1.65 | 5.26E−04 | 39057 | Welsh |
| ***PLEKHM1*** | Down | −11.15 | 0.005 | AB002354_at | Luo |
| ***PLEKHM2*** | Down | −1.13 | 4.87E−04 | 36150_at | Yu |

### Supplementary Table 4: APCB cohort

| Gene | Fold change | *P* value | Summary |
| --- | --- | --- | --- |
| *ARF6* | 1.23 | 2.78E−06 | **** |
| *ARL8A* | 1 | 0.934 | Ns |
| *ARL8B* | −1.08 | 0.00263 | **** |
| *DCTN1* | −1 | 0.962 | Ns |
| *DYNC1H1* | 1.01 | 0.355 | Ns |
| *DYNC1I1* | −1.37 | 5.04E−05 | **** |
| *KIF5B* | 1 | 0.94 | Ns |
| *KIF5C* | 1.07 | 0.408 | Ns |
| *KLC1* | −1.05 | 0.128 | Ns |
| *KLC2* | 1.02 | 0.514 | Ns |
| *MAPK8IP3* | −1.03 | 0.279 | Ns |
| *PIP4P1* | −1 | 0.983 | Ns |
| *PLEKHM1* | −1.09 | 0.00395 | **** |
| *PLEKHM2* | −1.01 | 0.802 | Ns |
| *SPAG9* | −1.1 | 8.79E−05 | **** |

### Supplementary Table 5: Expression of lysosome trafficking machinery filtered by Gleason pattern. Related to Figure 2A.

|  | **GP 3**  n = 201 | | | | **GP 4**  n = 250 | | | | **GP 5**  n = 49 | | | |
| --- | --- | --- | --- | --- | --- | --- | --- | --- | --- | --- | --- | --- |
| **Gene** |  | **Fold change** | ***P-*value** |  |  | **Fold change** | ***P -*value** |  |  | **Fold change** | ***P -*value** |  |
|  |  |  |  |  |  |  |  |  |  |  |  |  |
|  |  |  |  |  |  |  |  |  |  |  |  |  |
| ***KIF5C*** |  | 1.22 | 0.032 |  |  | 1.3 | 0.005 |  |  | 1.14 | 0.402 |  |
|  |  |  |  |  |  |  |  |  |  |  |  |  |
|  |  |  |  |  |  |  |  |  |  |  |  |  |
| ***ARF6*** |  | 1.31 | < 0.001 |  |  | 1.32 | < 0.001 |  |  | 1.29 | < 0.001 |  |
|  |  |  |  |  |  |  |  |  |  |  |  |  |
|  |  |  |  |  |  |  |  |  |  |  |  |  |
| ***DYNC1H1*** |  | 1.05 | 0.516 |  |  | 1.01 | 0.933 |  |  | 1.04 | 0.643 |  |
|  |  |  |  |  |  |  |  |  |  |  |  |  |
|  |  |  |  |  |  |  |  |  |  |  |  |  |
| ***KLC1*** |  | 1.26 | < 0.001 |  |  | 1.34 | < 0.001 |  |  | 1.5 | < 0.001 |  |
|  |  |  |  |  |  |  |  |  |  |  |  |  |
|  |  |  |  |  |  |  |  |  |  |  |  |  |
| ***PIP4P1*** |  | 1.03 | 0.416 |  |  | 1.12 | 0.005 |  |  | 1.18 | 0.003 |  |
|  |  |  |  |  |  |  |  |  |  |  |  |  |
|  |  |  |  |  |  |  |  |  |  |  |  |  |
| ***PLEKHM2*** |  | 1.04 | 0.349 |  |  | 1.1 | 0.006 |  |  | 1.18 | 0.004 |  |
|  |  |  |  |  |  |  |  |  |  |  |  |  |
|  |  |  |  |  |  |  |  |  |  |  |  |  |
| ***MAPK8IP3*** |  | 1.2 | 0.049 |  |  | 1.45 | < 0.001 |  |  | 1.72 | < 0.001 |  |
|  |  |  |  |  |  |  |  |  |  |  |  |  |
|  |  |  |  |  |  |  |  |  |  |  |  |  |
| ***ARL8A*** |  | 1.03 | 0.443 |  |  | 1.16 | < 0.001 |  |  | 1.33 | < 0.001 |  |
|  |  |  |  |  |  |  |  |  |  |  |  |  |
|  |  |  |  |  |  |  |  |  |  |  |  |  |
| ***KLC2*** |  | 1.17 | 0.002 |  |  | 1.32 | < 0.001 |  |  | 1.49 | < 0.001 |  |
|  |  |  |  |  |  |  |  |  |  |  |  |  |
|  |  |  |  |  |  |  |  |  |  |  |  |  |
| ***KIF5A*** |  | −2.26 | < 0.001 |  |  | −1.8 | < 0.001 |  |  | 1.48 | 0.105 |  |
|  |  |  |  |  |  |  |  |  |  |  |  |  |
|  |  |  |  |  |  |  |  |  |  |  |  |  |
| ***ARL8B*** |  | −1.25 | < 0.001 |  |  | −1.13 | 0.004 |  |  | −1.03 | 0.734 |  |
|  |  |  |  |  |  |  |  |  |  |  |  |  |
|  |  |  |  |  |  |  |  |  |  |  |  |  |
| ***KIF5B*** |  | −1.09 | 0.119 |  |  | −1.15 | 0.017 |  |  | −1.23 | 0.008 |  |
|  |  |  |  |  |  |  |  |  |  |  |  |  |
|  |  |  |  |  |  |  |  |  |  |  |  |  |
| ***DYNC1I1*** |  | −2.09 | < 0.001 |  |  | −2.52 | < 0.001 |  |  | −2.06 | < 0.001 |  |
|  |  |  |  |  |  |  |  |  |  |  |  |  |
|  |  |  |  |  |  |  |  |  |  |  |  |  |
| ***PLEKHM1*** |  | −1.22 | < 0.001 |  |  | −1.27 | < 0.001 |  |  | −1.22 | 0.01 |  |
|  |  |  |  |  |  |  |  |  |  |  |  |  |
|  |  |  |  |  |  |  |  |  |  |  |  |  |
| ***DCTN1*** |  | −1.11 | 0.073 |  |  | −1.12 | 0.035 |  |  | −1.04 | 0.652 |  |
|  |  |  |  |  |  |  |  |  |  |  |  |  |
|  |  |  |  |  |  |  |  |  |  |  |  |  |
| ***SPAG9*** |  | −1.3 | < 0.001 |  |  | −1.3 | < 0.001 |  |  | −1.16 | 0.064 |  |
|  |  |  |  |  |  |  |  |  |  |  |  |  |

### Supplementary Table 6a: P values of altered lysosome positioning in cells from over-expression of molecular machinery. Related to Figure 4.

| Protein | PNT1a | LNCaP | PC‑3 |
| --- | --- | --- | --- |
| ARL8B | 0.112 | **0.0159** | **0.018** |
| JIP3 | **0.038** | **0.060** | 0.597 |
| JIP4 | 0.105 | 0.909 | 0.756 |
| KIF5C | 0.144 | **0.0035** | 0.171 |
| KLC1 | 0.747 | 0.233 | 0.448 |
| KLC2 | 0.921 | 0.233 | 0.287 |
| PLEKHM2/SKIP | 0.200 | 0.796 | 0.155 |

### Supplementary Table 6b: P-Values of lysosome count

| Protein | PNT1a | LNCaP | PC‑3 |
| --- | --- | --- | --- |
| ARL8B | ns | Ns | Ns |
| JIP3 | 0.01 | Ns | Ns |
| JIP4 | 0.001 | Ns | Ns |
| KIF5C | Ns | Ns | Ns |
| KLC1 | 0.01 | 0.01 | Ns |
| KLC2 | 0.05 | Ns | Ns |
| PLEKHM2/ SKIP | ns | Ns | ns |

### Supplementary Table 6c: P-Values of cell ellipticity (Oblate)

| Protein | PNT1a | LNCaP | PC‑3 |
| --- | --- | --- | --- |
| ARL8B | ns | Ns | Ns |
| JIP3 | 0.01 | Ns | Ns |
| JIP4 | 0.001 | Ns | Ns |
| KIF5C | Ns | Ns | Ns |
| KLC1 | 0.01 | 0.01 | Ns |
| KLC2 | 0.05 | Ns | Ns |
| PLEKHM2/ SKIP | ns | Ns | ns |

### Supplementary Table 6d: P-Values from Ellipsoid Width

| Protein | PNT1a | LNCaP | PC‑3 |
| --- | --- | --- | --- |
| ARL8B | Ns | 0.05 | Ns |
| JIP3 | Ns | 0.05 | Ns |
| JIP4 | Ns | Ns | Ns |
| KIF5C | Ns | Ns | Ns |
| KLC1 | Ns | 0.01 | Ns |
| KLC2 | Ns | Ns | Ns |
| PLEKHM2/ SKIP | Ns | Ns | 0.05 |

### Supplementary Table 6e: Ellipsoid Length

| Protein | PNT1a | LNCaP | PC‑3 |
| --- | --- | --- | --- |
| ARL8B | 0.01 | Ns | 0.01 |
| JIP3 | Ns | 0.05 | Ns |
| JIP4 | Ns | 0.05 | Ns |
| KIF5C | Ns | Ns | 0.001 |
| KLC1 | Ns | 0.01 | 0.01 |
| KLC2 | Ns | Ns | 0.01 |
| PLEKHM2/ SKIP | Ns | Ns | Ns |

### Supplementary Table 7: Androgen receptor expression correlates to trafficking machinery (APCB RNASeq dataset)

| **AR vs.** | **r** | **95 % confidence interval** | **R squared** | ***P* (two-tailed)** | ***P* value summary** |
| --- | --- | --- | --- | --- | --- |
| KIF5C | 0.6208 | 0.4951 to 0.7211 | 0.3855 | <0.0001 | **** |
| DCTN3 | 0.5832 | 0.4493 to 0.6915 | 0.3402 | <0.0001 | **** |
| DYNC1LI1 | 0.5603 | 0.4217 to 0.6733 | 0.3139 | <0.0001 | **** |
| ALG2 | 0.5146 | 0.3674 to 0.6366 | 0.2648 | <0.0001 | **** |
| LAMTOR3 | 0.4936 | 0.3428 to 0.6196 | 0.2436 | <0.0001 | **** |
| KIF13A | 0.4753 | 0.3216 to 0.6047 | 0.2259 | <0.0001 | **** |
| DCTN4 | 0.4597 | 0.3035 to 0.5919 | 0.2114 | <0.0001 | **** |
| ARL8B | 0.4249 | 0.2637 to 0.5630 | 0.1805 | <0.0001 | **** |
| DCTN5 | 0.4244 | 0.2631 to 0.5626 | 0.1801 | <0.0001 | **** |
| SNAPIN | 0.4224 | 0.2609 to 0.5609 | 0.1784 | <0.0001 | **** |
| BLOC1S1 | 0.4213 | 0.2596 to 0.5600 | 0.1775 | <0.0001 | **** |
| BLOC1S2 | 0.3773 | 0.2102 to 0.5230 | 0.1424 | <0.0001 | **** |
| KIF1A | 0.375 | 0.2076 to 0.5211 | 0.1407 | <0.0001 | **** |
| KIF1BP | 0.3523 | 0.1824 to 0.5017 | 0.1241 | <0.0001 | **** |
| KIF1B | 0.3356 | 0.1640 to 0.4874 | 0.1126 | 0.0002 | *** |
| BICD1 | 0.3315 | 0.1596 to 0.4840 | 0.1099 | 0.0003 | *** |
| LAMTOR1 | 0.3015 | 0.1270 to 0.4580 | 0.09093 | 0.001 | *** |
| ARF6 | 0.2823 | 0.1062 to 0.4412 | 0.0797 | 0.002 | ** |
| DCTN6 | 0.2664 | 0.08919 to 0.4273 | 0.07097 | 0.0037 | ** |
| HOOK1 | 0.244 | 0.06534 to 0.4075 | 0.05953 | 0.008 | ** |
| LAMTOR2 | 0.2413 | 0.06248 to 0.4051 | 0.05821 | 0.0088 | ** |
| DCTN2 | 0.2204 | 0.04053 to 0.3865 | 0.04859 | 0.0169 | * |
| LAMTOR4 | 0.2134 | 0.03320 to 0.3802 | 0.04556 | 0.0209 | * |
| ARL8A | 0.2118 | 0.03152 to 0.3788 | 0.04488 | 0.0219 | * |
| KLC2 | 0.1849 | 0.003512 to 0.3546 | 0.0342 | 0.0459 | * |
| DYNC1H1 | 0.1837 | 0.002247 to 0.3535 | 0.03375 | 0.0474 | * |
| HTT | −0.2236 | −0.3894 to −0.04389 | 0.05002 | 0.0154 | * |
| KIF13B | −0.2291 | −0.3942 to −0.04962 | 0.05248 | 0.013 | * |
| SPAG9 | −0.2343 | −0.3989 to −0.05509 | 0.05489 | 0.011 | * |
| FYCO1 | −0.2662 | −0.4271 to −0.08892 | 0.07084 | 0.0037 | ** |
| RAB7B | −0.289 | −0.4471 to −0.1134 | 0.08353 | 0.0016 | ** |
| DYNC1I1 | −0.2972 | −0.4542 to −0.1223 | 0.08833 | 0.0011 | ** |
| ANK2 | −0.3203 | −0.4743 to −0.1474 | 0.1026 | 0.0004 | *** |
| PLEKHM1P1 | −0.3417 | −0.4926 to −0.1707 | 0.1167 | 0.0002 | *** |
| ZFYVE27 | −0.3503 | −0.5001 to −0.1803 | 0.1227 | 0.0001 | *** |
| PLEKHM1 | −0.3592 | −0.5076 to −0.1901 | 0.129 | <0.0001 | **** |
| MAPK8IP3 | −0.4026 | −0.5443 to −0.2385 | 0.1621 | <0.0001 | **** |
| PLEKHM2 | −0.4154 | −0.5551 to −0.2530 | 0.1726 | <0.0001 | **** |
| KIF16B | −0.4166 | −0.5561 to −0.2543 | 0.1735 | <0.0001 | **** |
| RILPL2 | −0.5245 | −0.6447 to −0.3791 | 0.2751 | <0.0001 | **** |

### Supplementary Table 8: R1881 treatment alters expression of lysosome trafficking machinery in LNCaP cells. Related to Figure 5D.

|  | **1 nM** | | | | **10 nM** | | | | **100 nM** | | | |
| --- | --- | --- | --- | --- | --- | --- | --- | --- | --- | --- | --- | --- |
| **Gene** |  | **Fold change** | ***P* value** |  |  | **Fold change** | ***P* value** |  |  | **Fold change** | ***P* value** |  |
|  |  |  |  |  |  |  |  |  |  |  |  |  |
|  |  |  |  |  |  |  |  |  |  |  |  |  |
| ***ARL8A*** |  | 1.26 | < 0.001 |  |  | 1.28 | < 0.001 |  |  | 1.26 | < 0.001 |  |
|  |  |  |  |  |  |  |  |  |  |  |  |  |
|  |  |  |  |  |  |  |  |  |  |  |  |  |
| ***ARL8B*** |  | 1.54 | < 0.001 |  |  | 1.56 | < 0.001 |  |  | 2.79 | < 0.001 |  |
|  |  |  |  |  |  |  |  |  |  |  |  |  |
|  |  |  |  |  |  |  |  |  |  |  |  |  |
| ***KIF5C*** |  | 2.55 | < 0.001 |  |  | 2.55 | < 0.001 |  |  | 1.80 | < 0.001 |  |
|  |  |  |  |  |  |  |  |  |  |  |  |  |
|  |  |  |  |  |  |  |  |  |  |  |  |  |
| ***KIF5B*** |  | −1.25 | < 0.001 |  |  | −1.23 | < 0.001 |  |  | −1.23 | < 0.001 |  |
|  |  |  |  |  |  |  |  |  |  |  |  |  |
|  |  |  |  |  |  |  |  |  |  |  |  |  |
| ***KLC1*** |  | 1.38 | < 0.001 |  |  | 1.43 | < 0.001 |  |  | 1.36 | < 0.001 |  |
|  |  |  |  |  |  |  |  |  |  |  |  |  |
|  |  |  |  |  |  |  |  |  |  |  |  |  |
| ***KLC2*** |  | 1.80 | 0.008 |  |  | 1.80 | 0.032 |  |  | 1.48 | 0.005 |  |
|  |  |  |  |  |  |  |  |  |  |  |  |  |
|  |  |  |  |  |  |  |  |  |  |  |  |  |
| ***PLEKHM1*** |  | 1.37 | < 0.001 |  |  | 1.47 | < 0.001 |  |  | 1.07 | < 0.001 |  |
|  |  |  |  |  |  |  |  |  |  |  |  |  |
|  |  |  |  |  |  |  |  |  |  |  |  |  |
| ***PLEKHM2*** |  | 1.06 | 0.188 |  |  | 1.09 | < 0.05 |  |  | 1.47 | 0.166 |  |
|  |  |  |  |  |  |  |  |  |  |  |  |  |
|  |  |  |  |  |  |  |  |  |  |  |  |  |
| ***ARF6*** |  | 1.33 | < 0.001 |  |  | 1.34 | < 0.001 |  |  | 1.06 | < 0.001 |  |
|  |  |  |  |  |  |  |  |  |  |  |  |  |
|  |  |  |  |  |  |  |  |  |  |  |  |  |
| ***DCTN1*** |  | −1.08 | < 0.01 |  |  | −1.09 | < 0.01 |  |  | −1.09 | < 0.01 |  |
|  |  |  |  |  |  |  |  |  |  |  |  |  |
|  |  |  |  |  |  |  |  |  |  |  |  |  |
| ***DYNC1H1*** |  | 1.23 | < 0.001 |  |  | 1.27 | < 0.001 |  |  | 1.54 | < 0.001 |  |
|  |  |  |  |  |  |  |  |  |  |  |  |  |
|  |  |  |  |  |  |  |  |  |  |  |  |  |
| ***SPAG9*** |  | −1.15 | < 0.001 |  |  | −1.14 | < 0.001 |  |  | −1.05 | 0.184 |  |
|  |  |  |  |  |  |  |  |  |  |  |  |  |
|  |  |  |  |  |  |  |  |  |  |  |  |  |
| ***MAPK8IP3*** |  | 1.06 | 0.264 |  |  | 1.13 | < 0.01 |  |  | 1.53 | 0.143 |  |
|  |  |  |  |  |  |  |  |  |  |  |  |  |

### Supplementary Table 9: P values for protein expression from R1881 treatment of LNCaP cells. Related to Figure 5E.

| **Protein** | **1nM** | **10 nM** | **100 nM** |
| --- | --- | --- | --- |
| **ARL8B** | < 0.0001 | < 0.0001 | < 0.0001 |
| **KIF5C** | < 0.05 | < 0.05 | < 0.05 |
| **KIF5B** | < 0.05 | Ns | Ns |
| **KLC1** | < 0.0001 | < 0.001 | < 0.0001 |
| **KLC2** | < 0.0001 | < 0.05 | < 0.001 |
| **PLEKHM1** | < 0.001 | < 0.05 | < 0.0001 |
| **SKIP (PLEKHM2)** | Ns | Ns | Ns |
| **ARF6** | Ns | < 0.001 | < 0.05 |
| **DCTN1** | Ns | Ns | < 0.01 |
| **DYNC1I1** | Ns | < 0.0001 | < 0.05 |
| **JIP3 (MAPK8IP3)** | Ns | Ns | Ns |
| **JIP4 (SPAG9)** | Ns | Ns | Ns |

***Supplementary Table 10: siRNA mediated knock-down affects cell migration. Related to Figure 6.***

| siRNA | Cell line | Change versus Scrambled | P Value |
| --- | --- | --- | --- |
| *ARL8A* | PNT1a | No change | Ns |
|  | PC‑3 | No change | Ns |
| *ARL8B* | PNT1a | No change | Ns |
|  | PC‑3 | No change | Ns |
| *KIF5A* | PNT1a | No change | Ns |
|  | PC‑3 | Increased | 0.01 |
| *KIF5B* | PNT1a | No change | Ns |
|  | PC‑3 | No change | Ns |
| *KIF5C* | PNT1a | No change | Ns |
|  | PC‑3 | Increased | 0.01 |
| *KLC1* | PNT1a | No change | Ns |
|  | PC‑3 | Decreased | 0.0001 |
| *KLC2* | PNT1a | No change | Ns |
|  | PC‑3 | No change | Ns |
| *PLEKHM1* | PNT1a | No change | Ns |
|  | PC‑3 | Decreased | 0.001 |
| *PLEKHM2* (SKIP) | PNT1a | No change | Ns |
|  | PC‑3 | No change | Ns |
| *ARF6* | PNT1a | Decreased | 0.0001 |
|  | PC‑3 | Increased | 0.001 |
| *DCTN1* | PNT1a | No change | Ns |
|  | PC‑3 | Increased | 0.01 |
| *DYNC1H1* | PNT1a | No change | Ns |
|  | PC‑3 | Decreased | 0.05 |
| *DYNC1I1* | PNT1a | Decreased | 0.001 |
|  | PC‑3 | Decreased | 0.01 |
| *MAPK8IP3* (JIP3) | PNT1a | Increased | 0.01 |
|  | PC‑3 | No change | Ns |
| *SPAG9* (JIP4) | PNT1a | Decreased | 0.001 |
|  | PC‑3 | Increased | 0.05 |
